# Enriched Environment Reduces Seizure Susceptibility via Entorhinal Cortex Circuit Augmented Adult Neurogenesis

**DOI:** 10.1002/advs.202410927

**Published:** 2024-10-22

**Authors:** Zhongxia Li, Liying Chen, Fan Fei, Wenqi Wang, Lin Yang, Yu Wang, Heming Cheng, Yingwei Xu, Cenglin Xu, Shuang Wang, Yan Gu, Feng Han, Zhong Chen, Yi Wang

**Affiliations:** ^1^ Key Laboratory of Neuropharmacology and Translational Medicine of Zhejiang Province School of Pharmaceutical Sciences Zhejiang Chinese Medical University Hangzhou Zhejiang 310053 China; ^2^ Zhejiang Rehabilitation Medical Center Department The Third Affiliated Hospital Zhejiang Chinese Medical University Hangzhou Zhejiang 310061 China; ^3^ Department of Pharmacy Sir Run Run Shaw Hospital School of Medicine Zhejiang University Hangzhou Zhejiang 310016 China; ^4^ Epilepsy Center The Second Affiliated Hospital & School of Basic Medical Sciences Zhejiang University Hangzhou Zhejiang 310027 China; ^5^ Key Laboratory of Cardiovascular & Cerebrovascular Medicine Drug Target and Drug Discovery Center School of Pharmacy Nanjing Medical University Nanjing Jiangsu 211166 China

**Keywords:** EC‐DG pathway, enriched environment, epilepsy, neurogenesis

## Abstract

Enriched environment (EE), characterized by multi‐sensory stimulation, represents a non‐invasive alternative for alleviating epileptic seizures. However, the mechanism by which EE exerts its therapeutic impact remains incompletely understood. Here, it is elucidated that EE mitigates seizure susceptibility through the augmentation of adult neurogenesis within the entorhinal cortex (EC) circuit. A substantial upregulation of adult hippocampal neurogenesis concomitant with a notable reduction in seizure susceptibility has been found following exposure to EE. EE‐enhanced adult‐born dentate granule cells (abDGCs) are functionally activated during seizure events. Importantly, the selective activation of abDGCs mimics the anti‐seizure effects observed with EE, while their inhibition negates these effects. Further, whole‐brain c‐Fos mapping demonstrates increased activity in DG‐projecting EC CaMKIIα^+^ neurons in response to EE. Crucially, EC CaMKIIα^+^ neurons exert bidirectional modulation over the proliferation and maturation of abDGCs that can activate local GABAergic interneurons; thus, they are essential components for the anti‐seizure effects mediated by EE. Collectively, this study provides compelling evidence regarding the circuit mechanisms underlying the effects of EE treatment on epileptic seizures, shedding light on the involvement of the EC‐DG circuit in augmenting the functionality of abDGCs. This may help for the translational application of EE for epilepsy management.

## Graphical abstract

In this study, Li et al reveal circuit mechanisms underlying the enriched environment (EE) treatment on epilepsy. EE recruits entorhinal cortex circuit, promoting the proliferation and maturation of adult‐born dentate granule cells (abDGCs), which can activate local GABAergic interneurons, thus resulting in seizure attenuation. This may hold therapeutic significance for the translational application of EE in the treatment of epilepsy.

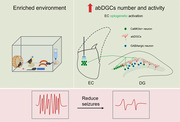

## 1 Introduction

Epilepsy is a common and serious neurological disorder characterized by recurrent seizures, affecting ≈70 million individuals across diverse age groups globally.^[^ ^1^ ^]^ While anti‐seizure drugs are often the first‐line treatment for seizure control, their efficacy is relatively limited and often accompanied by adverse events.^[^ ^2^ ^]^ Surgical removal of epileptogenic foci, an alternative for drug‐resistant cases, proves effective for only a minute fraction of patients due to the limitations like widespread distribution or localization within critical functional brain regions of epileptogenic foci. Furthermore, even after successful surgery, relapses and undesirable side effects persist among epilepsy patients.^[^ ^3^ ^]^ Altogether, these reflect the pressing needs of developing novel alternative treatments that could overcome the limitations of current therapeutic strategies.

The concept of an enriched environment (EE) encompasses a composite of multi‐sensory stimulation, social interaction, exercise, and emotional engagement.^[^ ^4^ ^]^ Noteworthy for its positive impact on brain development, cognitive function,^[^ ^5^ ^]^ and neuroprotective efficacy in various neurological disorders,^[^ ^5^, ^6^ ^]^ EE also has gained increased attention in recent years as a promising non‐invasive approach to ameliorating epileptic seizures. As early as 1999, the inhibitory effect of EE on seizures was first reported, demonstrating a lower incidence of seizures in rats reared in EE compared to those under standard conditions using a kainic acid (KA)‐induced epilepsy model.^[^ ^7^ ^]^ Subsequent studies have confirmed the anti‐seizure effects of EE across diverse epilepsy models, such as pilocarpine‐induced status epilepticus (SE),^[^ ^4b^ ^]^ amygdala kindling,^[^ ^8^ ^]^ pentylenetetrazol (PTZ)‐induced seizures^[^ ^9^ ^]^ and genetic models.^[^ ^10^ ^]^ What's more, EE treatment has been shown to help rescue aberrant structural changes in epilepsy,^[^ ^4^, ^11^ ^]^ including neuronal loss and mossy fiber sprouting in the epileptic hippocampus. Despite these encouraging outcomes, the precise mechanisms underlying the beneficial effects of EE in epilepsy remain incompletely elucidated.

EE treatment has been associated with the augmentation of adult hippocampal neurogenesis (AHN), characterized by increased proliferation of neural stem/progenitor cells under both physiological and epileptic conditions.^[^ ^4^, ^8^, ^12^ ^]^ It was proposed that AHN was related to the anti‐seizure effects of EE since EE was often found to influence seizures and AHN simultaneously.^[^ ^8^, ^13^ ^]^ In addition, alterations in the expression of various chemical mediators,^[^ ^10^, ^14^ ^]^ especially the upregulation of neurotrophins such as brain derived neurotrophic factor (BDNF) which is known for its role in regulating neurogenesis,^[^ ^15^ ^]^ have been identified as underlying mechanisms following EE treatment. Nonetheless, the direct evidence establishing the link between the therapeutic mechanism of EE in epilepsy and its modulation of neurogenesis remains elusive. Given the intricate and dynamic nature of AHN, involving intricate circuitry interactions, the question of whether the AHN‐enhancing and anti‐seizure effects of EE can be achieved through the modulation of specific neural circuits remains a crucial unresolved inquiry.

In this study, we confirmed a substantial upregulation of AHN concomitant with a notable reduction in seizure susceptibility following exposure to EE. EE enhanced adult‐born dentate granule cells (abDGCs) are sufficient and required for the anti‐seizure effects observed with EE. Furthermore, we revealed that the entorhinal cortex‐dentate gyrus (EC‐ DG) circuit not only augmented AHN but also mediated the anti‐seizure effects of EE. These findings provide important and direct evidence for the circuit mechanisms underlying the effects of EE on epileptic seizures, holding considerable therapeutic significance for the translational application of EE in the treatment of epilepsy.

## 2 Result

### 2.1 EE Enhances AHN and Reduces Seizure Susceptibility

**Figure 1:**
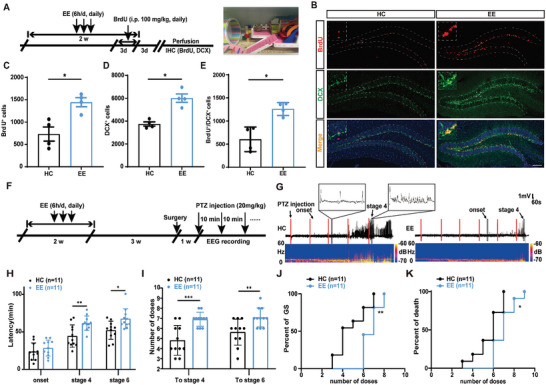
EE enhances AHN and reduces seizure susceptibility. A) Experiment scheme of using BrdU and DCX to label neuron proliferation in adult subgranular zone (SGZ) in the EE condition. BrdU was administered at the last 3 days of EE treatment. Perfusion was conducted 3 days post the last injection of BrdU and immunohistochemistry was then carried out. B) Representative images of BrdU and DCX labeling of EE treatment (bar = 100 µm). C–E) Proliferative activity in the SGZ was significantly increased for mice after EE treatment compared with housed in home cage (HC). n = 4 for each group, *p<0.05, compared with control; Mann Whitney test. F) Experiment scheme for examining the effect of EE on seizures susceptibility in a PTZ‐induced seizure model. G) Typical EEGs and power spectrograms recorded from the cortex in a PTZ induced seizure model. H–K) Effects of EE treatment on seizure susceptibility in a PTZ‐induced seizure model; H) latency to onset, stage 4, and stage 6; I) number of doses of PTZ required to reach stage 4 and stage 6; J) percent of mice reaching GS with increasing number of doses; K) percent of death with increasing number of doses. n = 11 for each group, *p<0.05, **p<0.01, ***p<0.001; for H, I Two‐way ANOVA followed by Sidak's test; for J, K, Log‐rank (Mantel‐Cox) tests were used to compare whole curves.

First, we aimed to see how EE affected AHN. Mice received EE treatment in a customized cage (47×30×30 cm^3^), which consists of tunnels, bridges, swings, stairs, a running wheel and several colored toys (wood, plastic, foam) similar to previous studies, for 14 d continuously (6 h d^−1^).^[^ ^4b^ ^]^ Then, we administered BrdU (100 mg kg^−1^ daily) during the last 3 days of EE to label mitotically active cells to characterize the level of cell proliferation in response to EE (**Figure** **1**A). Mice were perfused 3 days after the last injection of BrdU; immunohistochemistry of BrdU and doublecortin (DCX), marker of proliferating cells and immature neurons respectively, was carried out later to assess the level of AHN (Figure 1B). Our results showed that EE treatment led to a significant increase in the numbers of BrdU^+^ cells, DCX^+^ cells and BrdU^+^ DCX^+^ double‐labelled cells compared with controls (Figure 1C–E; Figure S1, Supporting Information). Moreover, we found that most of the BrdU expression were colocalized with DCX, while there are also a small proportion (≈12%) of BrdU‐positive but DCX‐negative cells, which are possibly proliferative radial glia‐like neural stem cells (rNSCs) and type 2a cells.^[^ ^16^ ^]^ In addition, we administered 100 mg kg^−1^ BrdU once daily at different timepoints to examine the dynamic daily change of cell proliferation in the DG and found that the number of BrdU‐positive cells significantly increased with longer EE treatment time, suggesting a cumulative effects of EE treatment (Figure S2, Supporting Information). Taken together, our results suggest EE has accumulative and stimulating effects on the proliferation of neural progenitors.

Next, to find out whether EE would influence epileptic seizures, we adopted a PTZ‐induced seizure model, which clinically resembles generalized tonic‐clonic seizures and is commonly used to test seizure susceptibility (Figure 1F).^[^ ^17^ ^]^ We found that EE treatment extended the latency to generalized seizures (GS) as well as increased the number of doses of PTZ (Figure 1H–K). Typical electroencephalograms (EEGs) and their corresponding power spectrums were shown in Figure 1G. Taken together, these results suggest that EE significantly enhance AHN while reducing seizure susceptibility.

### 2.2 Activation of abDGCs Mimics Anti‐Seizure Effect of EE

**Figure 2:**
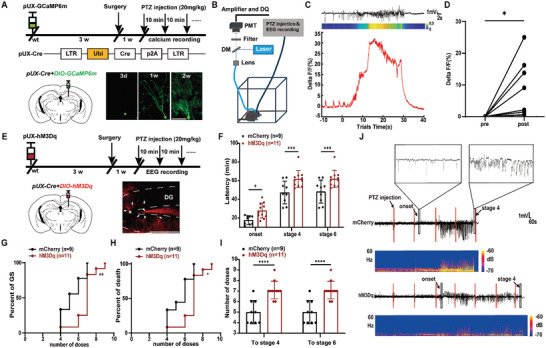
Chemogenetic activation of abDGCs reduces seizure susceptibility. A) Schematic diagram of the Ca^2+^ fiber photometry experiment. Fluorometric monitoring was carried out 4 weeks after the virus being injected to label abDGCs. Representative images of labelled abDGCs when they were 3d, 1w, 2w were shown separately. B) Configuration for fluorometric monitoring of Ca^2+^ signaling of abDGCs and simultaneous EEG recording during seizures. C) Representative trace showed the responses of GCaMP signals aligning with EEG recordings during seizures. D) The statistical value of △F/F_0_ was shown separately for each mouse (n = 8, *p<0.05, Paired t‐tests). E) Experiment scheme for chemogenetic activation of the abDGCs in a PTZ‐induced seizure model. 4 weeks after the injection of AAV‐EF1a‐DIO‐hM3Dq‐mCherry (at physiological states) to label newborn abDGCs, the first injection of PTZ was given to induce seizures. CNO was injected (1.0 mg kg^−1^, i.p.) 30 min before the first injection of PTZ to silence abDGCs. AAV‐EF1a‐DIO‐mCherry was used as control virus. A representative image of mCherry‐labelled abDGCs was shown, white arrows point to the labelled abDGCs. F–I) Effects of chemogenetic activation of abDGCs on seizures susceptibility in a PTZ‐induced seizure model; F) latency to onset, stage 4, and stage 6; G) percent of mice reaching GS with increasing number of doses; H) percent of death with increasing number of doses; I) number of doses of PTZ required to reach stage 4 and stage 6. n = 9 for mCherry, n = 11 for hM3Dq, *p<0.05, **p<0.01, ***p<0.001, ****p<0.0001; for F, I Two‐way ANOVA followed by Sidak's test; for G, H Log‐rank (Mantel‐Cox) tests were used to compare whole curves. J) Typical EEGs and power spectrograms recorded from the cortex during seizures in a PTZ‐induced seizure model.

Then, to investigate whether abDGCs were involved in anti‐seizure effects of EE, we first tested how abDGCs would be involved in epileptic seizures. We used Ca^2+^ fiber photometry to monitor the neural activity of abDGCs during PTZ‐induced seizures. We injected pUX‐Cre and AAV‐EF1a‐DIO‐GCaMP6m cocktail virus into the DG to label newly‐generated abDGCs (named pUX‐GCaMP6m mice, **Figure** **2**A). Before using the viral cocktail for experiments, we pretested them to ensure that the strategies we used to label newborn abDGCs were applicable (Figures S3 and S4, Supporting Information). As the morphological and physiological phenotypes of abDGCs gradually become mature at ≈4 weeks old,^[^ ^18^ ^]^ we measured changes in the GCaMP6m fluorescence of abDGCs by fiber photometry system when they were 4‐week‐old (Figure 2B) while EEG monitoring. Aligning the GCaMP6 signals with EEG, we found that epileptic seizures coincided with increased GCaMP6m fluorescence signal of abDGCs in pUX‐GCaMP6m mice (Figure 2C). Meanwhile, the maximum value of △F/F_0_ was shown separately for each trial (Figure 2D). These results reveal a seizure‐dependent activation of the neuronal activity of abDGCs, indicating that they are directly involved in epileptic seizures.

Next, to test the causal role of abDGCs in seizures, we applied chemogenetic strategies to achieve selective activation of abDGCs. We injected pUX‐Cre and AAV‐EF1a‐DIO‐hM3Dq‐mCherry cocktail virus into the DG to label abDGCs (named pUX‐hM3D mice, Figure 2E) and representative image of mCherry‐immunoreactive cells was shown. We found that in a PTZ‐induced seizure model, direct chemogenetic activation of 4‐week‐old abDGCs significantly increased the latency to seizure onset, stage 4 and stage 6 as well as the number of doses of PTZ required (Figure 2F–I). Typical EEGs and their corresponding power spectrums were shown in Figure 2J. These results suggest that activation of abDGCs reduces seizure susceptibility.

Similarly, we used optogenetic strategies to achieve selective activation of abDGCs. We injected pUX‐Cre and AAV‐EF1a‐DIO‐ChR2‐EYFP cocktail virus into the DG to label abDGCs (named pUX‐ChR2 mice, Figure S5A, Supporting Information). We found that although direct optogenetic activation of 4‐week‐old abDGCs further verify the anti‐seizure effect of abDGCs by significantly increasing the latency to stage 4 as well as the number of doses of PTZ required, it did not significantly increase the latency to stage 6 as the chemogenetic activation did (Figures S5B–F and S6, Supporting Information). The discrepancy of chemogenetic versus optogenetic stimulation of abDGCs could possibly be explained by the differences in activation pattern and area: chemogenetics may better resonate with the natural firing pattern of abDGCs^[^ ^19^ ^]^ and activate a relatively larger number of neurons than optogenetics. By contrast, surprisingly, chemogenetic inhibition of abDGCs had no effect on seizure susceptibility (Figure S7A–F, Supporting Information), which was an intriguing phenomenon. Firstly, abDGCs are normally firing at very low rates; thus, it may not be significant to inhibit abDGCs chemogenetically.^[^ ^20^ ^]^ Alternatively, feedforward excitation and inhibition are co‐existed within DG and the consequences of activation/inhibition of abDGCs may be a complex net effect resulting from different outputs.^[^ ^21^ ^]^ Altogether, these results indicate that selective activation of abDGCs is sufficient to play a protective role in epileptic seizures.

### 2.3 The abDGCs are Essential for the Anti‐Seizure Effect of EE

**Figure 3:**
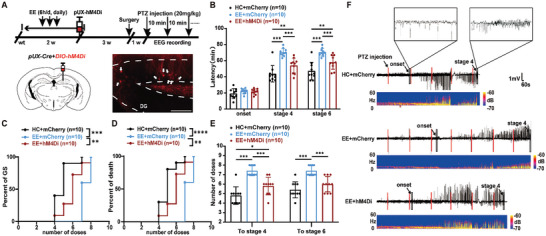
Inhibition of abDGCs abolishes the anti‐seizure effect of EE treatment. A) Experiment scheme for evaluating the effects of chemogenetically inhibiting the abDGCs generated during EE treatment on seizure susceptibility in a PTZ‐induced seizure model. Virus was injected at the last day of EE to label newborn abDGCs in response to EE. 4 weeks after the injection of AAV‐EF1a‐DIO‐hM4Di‐mCherry to label newborn abDGCs, the first injection of PTZ was given to induce seizures. CNO was injected (1.0 mg kg^−1^, i.p.) 30 min before the first injection of PTZ. A representative image of mCherry‐labelled abDGCs was shown, white arrows point to the labelled abDGCs. B–E) Effects of chemogenetic inhibition of abDGCs in EE on seizure susceptibility in a PTZ‐induced seizure model; B) latency to onset, stage 4 and stage 6; C) percent of mice reaching GS with increasing number of doses; D) percent of death with increasing number of doses; E) number of doses of PTZ required to stage 4 and stage 6. n = 10 for each group, *p<0.05, **p<0.01, ***p<0.001, ****p<0.0001, for B, E Two‐way ANOVA followed by Tukey's test; for C, D Log‐rank (Mantel‐Cox) tests were used to compare whole curves. F) Typical EEGs and power spectrograms recorded from the cortex during seizures in a PTZ induced seizure model.

Further, to test whether abDGCs were necessary for anti‐seizure effects of EE, chemogenetic tools were utilized to silence abDGCs generated at EE condition. Specifically, a virus cocktail of pAAV2/9‐EF1a‐DIO‐hM4Di‐mCherry together with pUX‐Cre was injected into the DG at the last day of EE to label abDGCs generated in EE (named pUX‐hM4D mice **Figure** **3**A). Behavioral tests were performed 4 weeks after the termination of EE exposure to allow virus expression and abDGCs maturation. Clozapine‐N‐oxide (CNO) (i.p. 1.0 mg kg^−1^) was infused 30 min before the first injection of PTZ to silence hM4D‐expressing abDGCs. Compared with home cage (HC)‐housed controls, EE‐exposed mice showed prolonged latency to seizure stage 4 and stage 6 as well as increased number of doses of PTZ in a PTZ‐induced seizure model; however, these effects were abolished in pUX‐hM4D mice (Figure 3B–F). Further analysis indicated that chemogenetic inhibition of abDGCs did not completely reverse the effects of EE, which may be due to the involvement of other cellular mechanisms or incomplete inhibition of the activity of all abDGCs generated during EE treatment.^[^ ^20^, ^21^, ^22^ ^]^ Overall, these results suggest that abDGCs generated at EE conditions are essential for anti‐seizure effects of EE.

### 2.4 DG‐Projecting EC Neurons Exhibit Increased Activity in EE

**Figure 4:**
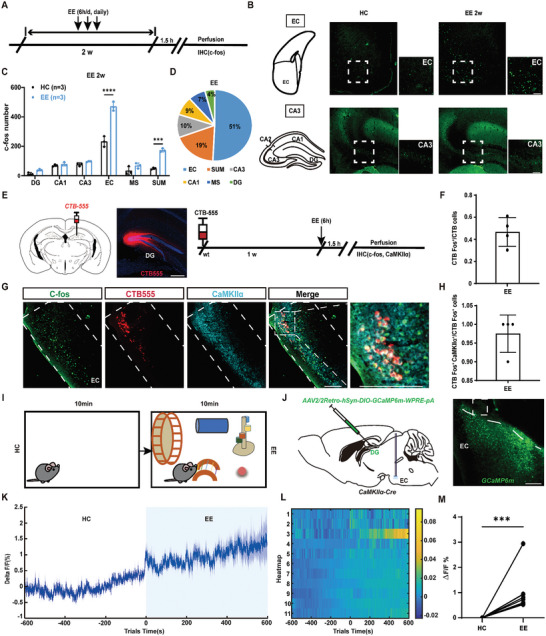
EE increases neural excitability of DG‐projecting EC CaMKIIα^+^ neurons. A) Experiment scheme of whole‐brain c‐Fos mapping in response to EE treatment. Mice were perfused 1.5 h post the termination of EE treatment. B) Representative images indicating the distribution of c‐Fos^+^ neurons in EC and CA3 (bar = 100 µm). C) The number of c‐Fos^+^ neurons in relevant brain regions of mice housed in HC (control) and after EE treatment. (n = 3 for each group ***p<0.001, ****p<0.0001, compared with control; Two‐way ANOVA followed by Sidak's test). D) Most (51%) of the c‐Fos^+^ neurons we identified were distributed in EC. E) Experiment scheme of c‐Fos labeling in response to EE in DG‐projecting EC neurons. CTB‐555 was injected into DG (bar = 100 µm) to visualize DG‐projecting neurons. F) The percentage of c‐Fos^+^ in DG‐projecting EC neurons (n = 4). G) Representative images indicating the distribution of c‐Fos^+^ DG‐projecting CaMKIIα^+^ neurons in EC (bar = 100 µm). H) The percentage of CaMKIIα^+^ in DG‐projecting EC c‐Fos^+^ neurons (n = 4). I) Diagram of fiber photometry recording of free‐moving mice in HC and EE for 10 min. J) Left: Diagram of calcium recording of DG‐projecting EC neurons. AAV2/2Retro‐DIO‐GCaMP6m‐WPRE‐pA was injected into the DG in *CaMKIIα‐Cre* mice to express GCaMP6 onto DG‐projecting CaMKIIα^+^ neurons and optical fiber was inserted into EC to collect calcium signals. Right: Representative image of GCaMP6m expression in EC neurons (bar = 100 µm). K) Mean fluorescence values of population activity of DG‐projecting EC neurons in the HC and EE. L) Heatmaps showing change of calcium signals. M) The statistical value of △F/F_0_ was shown for each mouse (n = 11, ***p<0.001, Paired t‐tests).

To identify the neural circuit mechanism involved in the EE‐enhanced AHN, mice were perfused 90 min post the end of EE (on day 14), then sliced and labelled with c‐Fos fluorescence by immunohistochemistry (**Figure** **4**A). The immediate early gene c‐Fos is a reliable marker of neuronal activity following a stimulus.^[^ ^23^ ^]^ We calculated c‐Fos^+^ neurons in the brain regions that were previously reported to be major inputs of the DG, including the CA1, CA3, EC, medial septum (MS) and supramammillary nucleus (SuM).^[^ ^24^ ^]^ We found that EE significantly increased c‐Fos expression in the EC and SuM as compared with mice housed in the home cage (Figure 4B,C). Meanwhile, we found that among the brain regions with increased c‐Fos expression, EC, which was generally accepted to be related to the sensory system,^[^ ^25^ ^]^ was the one with the highest proportion of increased c‐Fos expression (Figure 4D). More detailed analysis indicated that both lateral EC (LEC) and medial EC (MEC) showed a significant increase of c‐Fos expression after EE; however, no significant preference was detected between LEC and MEC (Figure S8, Supporting Information).

To further verify this result, CTB555 (Cholera Toxin Subunit B, CTB) was injected into the DG to label DG‐projecting neurons by retrograde tracing (named CTB555 mice). EE treatment is performed on day 7 post injection of CTB555 (Figure 4E). In the EC, ≈46% DG‐projecting EC neurons were positive for c‐Fos (Figure 4F). Then, we performed immunohistochemical co‐staining of c‐Fos with the markers for different types of neurons on brain slices of CTB555 mice (Figure 4G). We found that ≈97% of c‐Fos^+^ DG‐projecting EC neurons were positive for CaMKIIα (Figure 4H), indicating EE treatment mainly activate DG‐projecting EC CaMKIIα^+^ glutamatergic neurons. More detailed analysis indicated that the DG‐projecting EC CaMKIIα^+^ glutamatergic neurons were mainly distributed in layer II and layer III, which is consistent with previous studies.^[^ ^25c^ ^]^ Interestingly, we found that the distribution of c‐Fos^+^ DG‐projecting CaMKIIα^+^ neurons in the EC was layer‐dependent and predominantly distributed in layer II (Figure S9, Supporting Information).

Moreover, we recorded calcium dynamics in DG‐projecting EC neurons by Ca^2+^ fiber photometry in EE or home cage (Figure 4I). Specifically, we injected AAV2/2Retro‐DIO‐GCaMP6m into the DG in *CaMKIIα‐Cre* mice to label DG‐projecting EC CaMKIIα^+^ neurons (Figure 4J). Calcium activity in DG‐projecting EC CaMKIIα^+^ neurons was significantly increased when mice were in EE condition (Figure 4K–M). Overall, these results suggest that DG‐projecting EC CaMKIIα^+^ neurons are highly responsive to EE with increased activity, indicating that they may actively participate in the effects of EE‐enhanced AHN.

### 2.5 EC CaMKIIα^+^ Neurons Bi‐Directionally Modulate the Production and Morphological Maturation of abDGCs

**Figure 5:**
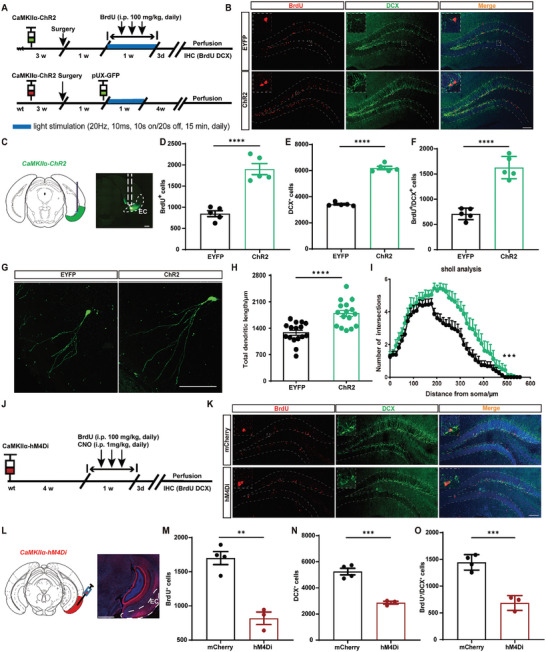
Chronic patterned optogenetic activation of EC CaMKIIα^+^ neurons increases proliferating abDGCs with improved developmental properties. A) Experiment scheme for optogenetic stimulation of EC CaMKIIα^+^ neurons. Blue light stimulation was given daily for 7 continuous days and the parameter was: 473 nm, 20 Hz, 10 ms pulse^−1^, 10s on/ 20s off, 5 mW and 15 min. Upper: using BrdU and DCX to label neuron proliferation in adult SGZ and perfusion was performed 3 days post the optogenetic stimulation. Lower: using pUX‐GFP virus to visualize abDGCs morphology in adult SGZ. Perfusion was performed 4w post the optogenetic stimulation to allow the morphological maturation of abDGCs. B) Representative images of BrdU^+^ DCX^+^ cells in the DG (bar = 100 µm). C) Histochemical verification of ChR2 (green) expression in the EC (bar = 100 µm). D–F) Proliferative activity in the SGZ was significantly increased after optogenetic stimulation of EC CaMKIIα^+^ neurons (n = 5 for each group **** p<0.0001, compared with control; Paired t‐tests). G) Representative confocal images of GFP‐immunoreactive cells (bar = 100 µm). H) abDGCs showed longer total dendritic length after optogenetic stimulation of EC CaMKIIα^+^ neurons (n = 4 for each group with 4 typical neurons considered for each animal, ****p<0.0001, compared with control; Student's t‐tests). I) Sholl analysis of abDGCs (n = 4 for each group ***p<0.001, compared with control; Student's t tests). J) Experiment scheme for chemogenetic inhibition of EC CaMKIIα^+^ neurons in mice for 7 continuous days. 4 weeks after virus injection, CNO (i.p. 1.0 mg kg^−1^) was given daily with BrdU (i.p. 100 mg kg^−1^) infused 30 min later. Perfusion was performed 3 days post the completion of chemogenetic inhibition. K) Representative images of BrdU^+^ DCX^+^ cells in the DG (bar = 100 µm). L) Immunostaining of hM4Di (red) expression in the EC (bar = 100 µm). M–O) Proliferative activity in the SGZ was significantly decreased after chemogenetic inhibition of EC CaMKIIα^+^ neurons (n = 4 for mCherry, n = 3 for hM4Di, **p<0.01, *** p<0.001, Student's t‐tests).

Then, we tested whether long‐term activation of EC CaMKIIα^+^ neurons were sufficient to enhance AHN. AAV‐CaMKIIα‐ChR2‐EYFP was injected into the EC, along with chronic EC stimulation following a 7‐day patterned light paradigm (**Figure** **5**A). Optogenetic activation of EC CaMKIIα^+^ neurons significantly increased the numbers of BrdU^+^ and DCX^+^ cells, along with an increased number of BrdU^+^ DCX^+^ cells compared with controls, suggesting that activation of EC CaMKIIα^+^ neurons promoted the proliferation of abDGCs (Figure 5B–F). Meanwhile, optogenetic activation of EC CaMKIIα^+^ neurons increased dendritic growth of 4w‐abDGCs compared with controls, indicated by the increased total dendritic length and the intersections of their dendrites (Figure 5G–I). These results indicated that EC stimulation facilitated the proliferation as well as the morphological maturation of abDGCs. On the contrary, we further used chemogenetic methods to inhibit EC CaMKIIα^+^ neurons by injecting AAV‐CaMKIIα‐hM4Di‐mCherry into the EC (Figure 5J). We found that EC‐inhibited mice exhibited significantly reduced numbers of BrdU^+^ and DCX^+^ cells, along with reduced numbers of BrdU^+^ DCX^+^ cells compared with controls (Figure 5K–O). In summary, these above results demonstrate that EC CaMKIIα^+^ neurons bi‐directionally modulate the production and morphological maturation of abDGCs. Notably, chronic patterned activation of EC neurons can mimic the functional activation of EE on EC CaMKIIα^+^ neurons as well as its enhancement on AHN.

### 2.6 EC CaMKIIα‐DG Neural Circuit is Required for the Effect of EE Treatment on AHN and Seizure Susceptibility

**Figure 6:**
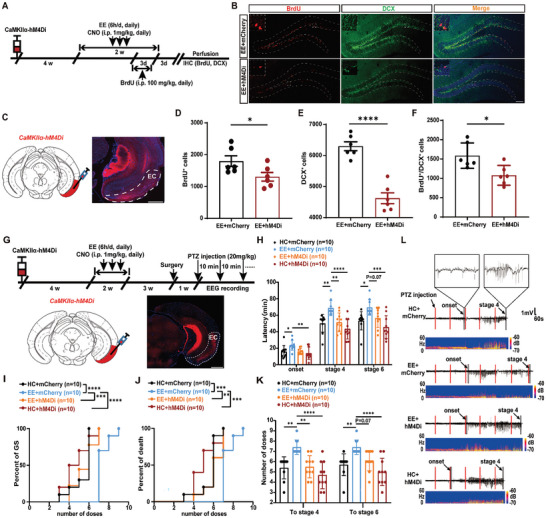
Inhibition of EC abolishes the effect of EE treatment in enhancing AHN and reducing seizure susceptibility. A) Experiment scheme for examining the effect of chemogenetically inhibiting EC CaMKIIα^+^ neurons on AHN during EE treatment. The treatment of EE was initiated 4 weeks after the virus injection to allow sufficient expression of pAAV2/8‐CaMKIIα‐hM4D(Gi)‐mCherry‐3xFlag‐WPRE. BrdU (i.p. 100 mg kg^−1^, daily) was administered at the last three days of EE treatment and CNO (i.p. 1.0 mg kg^−1^, daily) was infused 30 min before everyday EE for 14 consecutive days. B) Representative images of BrdU^+^ DCX^+^ cells in the DG (bar = 100 µm). C) Immunostaining of hM4Di (red) expression in the EC (bar = 100 µm). D–F) Effects of chemogenetic inhibition of EC CaMKIIα^+^ neurons in EE on AHN (n = 6 for each group *p<0.05, ****p<0.0001, compared with control; Student's t‐tests). G) Experiment scheme for chemogenetic inhibition of EC CaMKIIα^+^ neurons during EE treatment in a PTZ‐induced seizure model. CNO was administrated for 14 days during EE treatment (30 min before EE, i.p. 1.0 mg kg^−1^, daily). Immunostaining of hM4Di (red) expression in the EC (bar = 100 µm). H–K) Effects of chemogenetic inhibition of EC CaMKIIα^+^ neurons in EE on seizure susceptibility in a PTZ‐induced seizure model; H) latency to onset, stage 4 and stage 6; I) percent of mice reaching GS with increasing number of doses; J) percent of death with increasing number of doses; K) number of doses to stage 4 and stage 6. n = 10 for each group; for H,K Two‐way ANOVA followed by Tukey's test; for I, J Log‐rank (Mantel‐Cox) tests were used to compare whole curves. L) Typical EEGs and power spectrograms recorded from the cortex during seizures in a PTZ‐induced seizure model.

Further, we sought to address whether EC^CaMKIIα^‐DG neural circuit was required for EE‐induced effects on AHN and epilepsy. Toward this direction, EC CaMKIIα^+^ neurons were inhibited by injection of AAVs expressing CaMKIIα‐hM4Di into the EC and daily infusion of CNO 30 min before EE treatment (named EC‐inhibited mice, **Figure** **6**A). BrdU was administered at the last 3 days of EE, then perfusion and immunohistochemistry were carried out. Increased abDGCs were found in EE‐exposed mice as compared with home cage‐housed controls; however, these EE‐induced AHN‐upregulating effects were abolished in EC‐inhibited mice (Figure 6B–F). These results suggest that EC CaMKIIα^+^ neurons are essential for EE‐induced production and maturation of abDGCs.

To further determine the downstream circuit mechanisms of EC CaMKIIα^+^ neurons, first, we have utilized mWGA‐mCherry viral tracing^[^ ^26^ ^]^ to further determine the exact downstream targets of EC^CaMKIIα^‐DG circuit within the DG. We found that mCherry‐labelled cells were distributed widely in the hilus and GCL of DG. To investigate the cellular identity of the DG cells receiving afferents from EC CaMKIIα^+^ neurons, we performed immunohistochemistry and found that most mCherry‐labelled were CAMKIIα‐positive cells (91.51%), while GABA‐positive cells (5.13%) and DCX‐positive cells (2.61%) also account for a small proportion (Figure S10, Supporting Information), indicating direct connections between EC CaMKIIα^+^ neurons and DCX^+^ newborn cells, mature glutamatergic and GABAergic neurons. Further, we took advantage of the pharmacological methods to evaluate the association between the effects of EC CaMKIIα^+^ neurons on AHN with local glutamatergic and GABAergic transmission within DG. We applied glutamate receptor antagonists (intra‐DG injection, CNQX 10 µM plus D‐AP5 25 µM, 0.5 µL) or the GABA_A_ receptor antagonist bicuculline (intra‐DG injection, 5 µM, 0.5 µL) to separately block the glutamatergic or GABAergic neurotransmission while optogenetically activating EC CaMKIIα^+^ neurons. We found that the EC CaMKIIα^+^ neurons‐induced AHN‐upregulating effects were abolished by either local application of glutamate receptor antagonists or GABA_A_ receptor antagonist (Figure S11, Supporting Information). Altogether, these data indicate that the activation of EC CaMKIIα^+^ neurons may facilitate neurogenesis through both local glutamatergic and GABAergic transmission. As the state of NSCs and the process of AHN are regulated by DG local neurons, including mossy cells, mature granule cells (mGCs), interneurons, and microenvironment of the hippocampal neurogenic niche etc., our results showed that EC^CaMKIIα^‐DG circuit possibly recruit a combination of various downstream target‐cells (local GABAergic interneurons, mossy cells, mGCs and DCX^+^ newborn abDGCs) to exert their general effects on AHN and seizures, both directly and indirectly.

Next, we examined whether EE‐induced anti‐seizure effects depended on EC CaMKIIα^+^ neurons. CNO was infused following the same paradigm as was described above to inhibit EC^CaMKIIα^‐DG neural circuit and behavioral tests were performed 4 weeks after EE exposure (Figure 6G). EE‐housed mice showed reduced seizure susceptibility, as compared with home cage‐housed controls; however, these effects of EE were abolished in EC‐inhibited mice (Figure 6H–L). Interestingly, no significant changes were observed when comparing mice with intact EC and inhibited EC housed in home cage, suggesting that inhibition of EC alone did not significantly affect seizure susceptibility under the baseline condition. Together, these results suggest that EC CaMKIIα^+^ neurons are required for seizure‐modulating effects of EE. As was mentioned above, SuM was the brain region with the second highest percentage of c‐Fos expression increase after EE treatment; however, we found that it was not required for seizure‐modulating effects of EE (Figure S12, Supporting Information). Concerning that activation of abDGCs born under physiological conditions helps reduce susceptibility to seizures (Figure 2), it is natural for us to hypothesize that CaMKIIα^+^ neurons of EC may reduce the seizure susceptibility through activating the “protective” abDGCs.

### 2.7 The abDGCs Reduce Seizure Susceptibility via Local GABAergic Neurons

Alteration in the excitatory/inhibitory neuronal balance is generally believed to be the underlying mechanism of epileptic seizure. GABAergic interneurons are regarded as the primary inhibitory neurons and optogenetic excitation of GABAergic interneurons has been reported to achieve seizure suppression.^[^ ^27^ ^]^ Previous evidence suggests abDGCs make direct synaptic contacts through mossy fibers onto CA3 pyramidal cells and DG/hilar/CA3 interneurons,^[^ ^18^, ^28^ ^]^ which may provide feedback inhibition onto DG mGCs.^[^ ^28^, ^29^ ^]^ Consequently, we hypothesized that abDGCs might exert its anti‐seizure effects by mediating local DG GABAergic interneurons. To examine this hypothesis, initially, using immunohistochemistry, we examined c‐Fos levels in the mGCs and local GABAergic interneurons during PTZ‐induced seizures. We found that selectively optogenetic activation of abDGCs significantly reduced c‐Fos levels in mGCs which are labelled by Prox1^[^ ^30^ ^]^ while increasing c‐Fos levels in GABAergic interneurons compared to the control group (Figure S13, Supporting Information).

**Figure 7:**
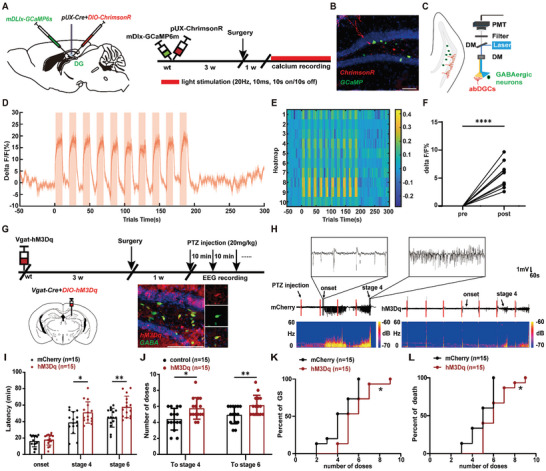
The abDGCs activate local anti‐seizure GABAergic neurons. A) Diagram of calcium recording of GABAergic neurons. AAV2/9‐mDlx‐GCaMP6s‐WPRE‐pA, AAV2/9‐FLEX‐ChrimsonR and pUX‐Cre cocktail virus were injected into DG. The parameter of red‐light stimulation is: 635 nm, 20 Hz, 10s on/off, 5 mW. B) Histochemical verification of GCaMP6m (green) and ChrimsonR (red) expression in the DG (bar = 100 µm). C) Configuration for fluorometric monitoring of Ca^2+^ signaling of GABAergic neurons. D) Mean fluorescence values showed that optogenetic activation of abDGCs (10s on‐off, 635 nm) increased Ca^2+^ level in local GABAergic neurons reliably. E) Heatmaps showing calcium signals. F) The statistical value of △F/F_0_ was shown for each mouse (n = 10, ****p<0.0001, Paired t‐tests). G) Experiment scheme for chemogenetic activation of GABAergic neurons in a PTZ‐induced seizure model. CNO was injected (1.0 mg kg^−1^, i.p.) 30 min before the first injection of PTZ. Histochemical verification of hM3D‐expressing GABAergic interneurons and double immunostaining of GABA (green) and hM3D (red) was shown (bar = 100 µm). H) Typical EEGs and power spectrograms recorded from the cortex during seizures in a PTZ induced seizure model. I–L) Effects of chemogenetic activation of GABAergic interneurons on seizure susceptibility; I) latency to onset, stage 4 and stage 6; J) number of doses to stage 4 and stage 6; K) percent of mice reaching GS with increasing number of doses; L) percent of death with increasing number of doses. n = 15 for each group, *p<0.05, **p<0.01; for I, J Two‐way ANOVA followed by Sidak's test were used; for K, L Log‐rank (Mantel‐Cox) tests were used to compare whole curves.

Further, we took advantage of in vivo calcium recording by fiber photometer to evaluate the functional connection between abDGCs and local GABAergic interneurons. We injected pUX‐Cre and AAV‐hSyn‐FLEX‐ChrimsonR‐tdTomato viral cocktail to label abDGCs. The ChrimsonR‐expressed abDGCs can be functionally activated by the red light.^[^ ^31^ ^]^ Meanwhile, we also injected AAV‐mDlx‐GCaMP6s‐WPRE‐pA to label GABAergic interneurons (**Figure** **7**A–C). We found that optogenetic activation of abDGCs (10s on‐off) largely increased Ca^2+^ level in the local GABAergic neurons reliably (Figure 7D–F), indicating the excitatory connections between abDGCs and GABAergic neurons. Moreover, chemogenetic activation of GABAergic neurons significantly increased the latency to seizure stage 4 and stage 6 as well as the number of doses of PTZ (Figure 7G–L), suggesting activation of DG GABAergic neurons reduced seizure susceptibility, which mimic the anti‐seizure effects of EE. Altogether, these results indicate the functional connection between abDGCs and local GABAergic neurons, providing possible explanations for the “protective” role of abDGCs generated under EE conditions.

## 3 Discussion

EE treatment represents a promising non‐invasive therapeutic option for individuals afflicted with epilepsy and concurrent psychiatric comorbidities, potentially complementing traditional medication regimens in the future. However, the precise underlying mechanisms of EE treatment remain elusive. Here, we verified that EE significantly upregulated AHN and concurrently reduced seizure susceptibility. Importantly, we ascertained that selective activation of abDGCs mimicked anti‐seizure effects observed with EE, whereas inhibition of them abolished these effects. This compelling evidence supports the statement that abDGCs mediate the therapeutic efficacy of EE. Although the level of AHN is relatively low, its pivotal role in central nervous system diseases, including epilepsy, cannot be underestimated.^[^ ^15^, ^32^ ^]^ The nonpharmacologic and non‐invasive nature of EE treatment positions it as an ideal choice for achieving enduring anti‐seizure effects through the modulation of abDGCs. Next, we investigated potential circuitry mechanisms underlying the anti‐seizure effects of EE treatment. Notably, Li et al. recently proposed a specific neural circuit (SuM‐DG) that can be manipulated to enhance AHN and highlighted the contribution of these circuit‐modified abDGCs in behavioral regulation, including memory, anxiety, and AD‐related deficits.^[^ ^33^ ^]^ However, the anti‐seizure effects were not mentioned in their studies, nor did the underlying mechanisms of EE. In our study, whole‐brain c‐Fos mapping revealed that EC, a region conventionally associated with the sensory system,^[^ ^25a,b^ ^]^ as the brain region exhibiting the highest proportion of increased c‐Fos in response to EE. Meanwhile, Ca^2+^ fiber photometry recording demonstrated heightened activity in DG‐projecting EC CaMKIIα^+^ neurons under EE conditions. Furthermore, we found that chronic activation of EC CaMKIIα^+^ neurons in a specified 7‐day patterned light paradigm bi‐directionally modulated the proliferation and enhanced the maturity of abDGCs, which were required for the effects of EE on seizure susceptibility. In addition, the exact targets of EC CaMKIIα‐DG circuit within the DG were also determined. Although EC predominantly recruits excitatory CAMKIIα‐positive cells, with the unique cellular and network properties of “dentate gate” and the increase of “protective” abDGCs,^[^ ^19^, ^20^, ^28^, ^34^ ^]^ chronic EC activation leads to reduced seizure susceptibility ultimately, mimicking the effects of EE. Consequently, our findings provide pivotal insights into the circuitry mechanisms underlying EE‐induced neurogenesis, while shedding light on the potential circuitry mechanisms underlying anti‐seizure effects of EE treatment. Previous studies have elucidated that the anti‐seizure effects of EE across diverse epilepsy models.^[^ ^4^, ^7^, ^8^, ^9^, ^10^, ^11^ ^]^ Thus, we believe these findings in our study can be largely generalized to other types of seizure models. However, more comprehensive and thorough studies are still in need to optimize the time and specific parameters of EE treatment.

Interestingly, conflicting results have been reported^[^ ^35^ ^]^ regarding the roles of abDGCs in epilepsy. Previous studies demonstrated that DG serve as a “gate” that guards the hippocampus from seizures initiated by neocortical activity.^[^ ^36^ ^]^ When AHN was blocked in rodents, hippocampal excitability was enhanced^[^ ^37^ ^]^ and the severity of SE induced by KA and pilocarpine was increased.^[^ ^35^, ^38^ ^]^ Correspondingly, in our study, the activation of abDGCs under EE demonstrated a reduction in seizure susceptibility, playing a protective role through the activation of local GABAergic interneurons. Conversely, there were studies suggesting a pro‐epileptic role of abDGCs.^[^ ^35^, ^39^ ^]^ Using two well‐established models of TLE (hippocampal kindling and KA induced SE model), our recently published work emphasized the importance of abDGCs generated acutely after seizures, demonstrating their direct involvement in seizure maintenance. Meanwhile, we provided direct evidence for the functional heterogeneity of abDGCs generated at different timepoints relative to seizures.^[^ ^40^ ^]^ In this study, however, we concentrated on the function of abDGCs generated in non‐epileptic states and specially in EE condition, revealing their protective role in seizure susceptibility, provided further evidence of functional heterogeneity of abDGCs to reconcile the above‐mentioned seemingly contradictory findings. Taken together, our results attach great importance to the temporal dynamics of abDGCs in epilepsy, with abDGCs generated at distinct time points relative to seizures playing diverse roles in the epileptic process. This heterogeneity may be related to the alterations in the microenvironment corresponding to different stages of the disease. Ultimately, our findings propose that EE fosters the generation of relatively normal and healthy abDGCs, imparting a protective effect on epilepsy by enhancing the activity of DG‐projecting EC neurons.

On the other hand, we found that the in vivo Ca^2+^ fiber photometry recording revealed an increase in GCaMP6m fluorescence signals of abDGCs while activating them mimicked the anti‐seizure effects of EE, which was an interesting and seeming conflictive observation. The activation of seizure‐protective of abDGCs can be regarded as one of the inherent compensatory mechanisms to protect the brain from toxic hyperexcitability.^[^ ^41^ ^]^ Notably, in our previous study,^[^ ^40^ ^]^ activation of the abDGCs generated acutely (3d) post being fully kindled were found to aggravate seizures; however, for them, decreased GCaMP6m fluorescence signals were found coincided with epileptic seizures. Taken together, abDGCs are heterogeneous populations and their activity during seizures are fine‐tuned to facilitate their compensatory responsibilities.

In conclusion, our study elucidates that the EC‐DG circuit, by augmenting adult neurogenesis, mediates the anti‐seizure effects of EE, thereby providing direct evidence regarding the circuit mechanisms underlying EE's impact on seizure susceptibility. These findings may hold therapeutic significance for the translational application of EE in the treatment of epilepsy.

## 4 Experimental Section

### Animals

This study examined male mice because male animals exhibited less variability in phenotype. In this experiment, C57BL/6 Wild type (RRID: IMSR_JAX:000664, male, 8–9 weeks old) mice were mainly used and purchased from SLAC Laboratory Animal Centre (Shanghai, China). *CaMKIIα‐Cre* (Stock number 0 0 5359, 8–9 weeks old) mice were used and genotyped in line with the protocols provided by the Jackson Laboratory. All animals were raised in animal houses on a 12 h‐light/dark circle with SPF standard conditions (22 ± 2 °C, relative humidity 50% ± 10%). Food and water were provided ad libitum. All procedures complied with the standards of the Institutional Animal Care and was approved by the ethical committee of Zhejiang Chinese Medical University and Zhejiang University (No.16804). Animal studies were reported in compliance with ARRIE guidelines.

### Enriched Environment

Mice were placed in EE for 14 d. An EE cage (47×30×30 cm^3^) consists of a running wheel, tunnels, bridges, swings, stairs, and several colored toys (wood, plastic, foam). Similar to previous studies, mice were kept in EE 6 h day^−1^ for continuous 14 days.^[^ ^4b^ ^]^ Mice had free access to food and water during EE exposure. The cage was placed in a climate‐controlled room on a 12 h light/dark cycle. The cage contained no more than 7 mice at a time. All toys and food stations were reorganized every day to promote exploration and ensure the novelty of the environment.

### Virus

The pUX‐Cre constructs were obtained from Yan Gu (Zhejiang University), produced in his own laboratory as their previous studies,^[^ ^18^, ^21^, ^42^ ^]^ using a murine leukemia retroviral vector to express Cre recombinase. By combining it with the use of Cre‐inducible recombinant adeno‐associated virus (AAV) vector containing viruses, selective expression can be conferred to proliferating cells. For fluorometric monitoring of abDGCs, a viral cocktail (1:1, 0.4 µL) of Cre‐inducible recombinant AAV vector containing GCaMP6m (rAAV2/9‐EF1a‐DIO‐GCaMP6m‐WPRE, 6.70*10^12^ particles mL^−1^) and pUX‐Cre (retrovirus) were injected stereotactically into the DG (AP, ‐2.0 mm; ML, ‐1.2 mm; DV, ‐2.1 mm). For fluorometric monitoring of GABAergic neurons and DG projecting EC CaMKIIα‐positive neurons, AAV2/9‐mDlx‐GCaMP6s‐WPRE‐pA (1.97*10^13^ particles mL^−1^) and AAV2/2Retro‐hSyn‐DIO‐GCaMP6m‐WPRE‐pA (1.65*10^13^ particles mL^−1^, 0.3 µL) were separately injected stereotactically into the DG. For selectively optogenetic activation of abDGCs, a viral cocktail (1:1, 0.6 µL) of AAV2/9‐hEF1a‐DIO‐hChR2(H134R)‐EYFP‐WPRE‐pA (1.69*10^13^ particles mL^−1^) or AAV2/9‐hSyn‐FLEX‐ChrimsonR‐tdTomato‐WPRE‐pA (1.9*10^13^ particles mL^−1^) mixed with pUX‐Cre were stereotactically injected into the DG. To chemogenetically activate or inhibit abDGCs, AAV2/9‐hEF1a‐DIO‐hM3D(Gq)‐mCherry‐WPRE‐pA (6.80*10^12^ particles mL^−1^, 0.4 µL) or pAAV2/9‐EF1a‐DIO‐hM4Di‐mCherry (3.46*10^12^ particles mL^−1^, 0.4 µL) was injected simultaneously with pUX‐Cre. To selectively modulate EC CaMKIIα^+^ neurons, mice were stereotactically injected with pAAV2/9‐CaMKIIα‐hChR2(H134R)‐EYFP (7.90*10^12^ particles mL^−1^, 0.3 µL) or pAAV2/8‐CaMKIIα‐hM4D(Gi)‐mCherry‐3xFlag‐WPRE (1.26*10^13^ particles mL^−1^, 0.3 µL) into the EC (AP: ‐4.0 mm, ML: ‐4.1 mm, DV: ‐4.8 mm) or SuM (AP, −2.4 mm; ML, ‐0.6 mm; DV, −4.85 mm). AAV2/9‐hEF1a‐DIO‐EYFP‐WPRE‐pA (2.60*1012 particles mL^−1^) and AAV2/9‐EF1a‐DIO‐mCherry (5.09*1012 particles mL^−1^) were used as control viruses. To selectively modulate GABAergic interneurons, a viral cocktail (1:1, 0.6 µL) of AAV2/9‐hEF1a‐DIO‐hM3D(Gq)‐mCherry‐ER2‐WPRE‐pA (6.80*1012 particles mL^−1^) mixed with rAAV2/9‐VGAT1‐Cre‐EGFP‐WPRE‐hGH‐pA (5.22*10^12^ particles mL^−1^) was used. AAV2/9‐EF1a‐DIO‐mCherry (5.09*10^12^ particles mL^−1^) was used as control viruses. rAAV2/9‐EF1a‐DIO‐GCaMP6m‐WPRE, AAV2/9‐hEF1a‐DIO‐hChR2(H134R)‐EYFP‐WPRE‐pA, pAAV2/9‐CaMKIIα‐hChR2(H134R)‐EYFP and pAAV2/8‐CaMKIIα‐hM4D(Gi)‐mCherry‐3xFlag‐WPRE were purchased from ObiO Technolog Corp., ltd. (Shanghai, China). AAV2/9‐hEF1a‐DIO‐EYFP‐WPRE‐pA, AAV2/9‐mDlx‐GcaMP6s‐WPRE‐pA, AAV2/2Retro‐hSyn‐DIO‐GCaMP6m‐WPRE‐pA, AAV2/9‐hSyn‐FLEX‐ChrimsonR‐tdTomato‐WPRE‐pA, AAV2/9‐hEF1a‐DIO‐hM3D(Gq)‐mCherry‐WPRE‐pA and pAAV2/9‐EF1a‐DIO‐hM4Di‐mCherry were purchased from Taitool Bioscience Co., ltd. (Shanghai, China). AAV2/9‐EF1a‐DIO‐mCherry and rAAV2/9‐VGAT1‐Cre‐EGFP‐WPRE‐pA were purchased from the Brain VTA Co., Ltd. (Wuhan, China). rAAV‐CAG‐DIO‐mWGA‐mCherry were purchased from Brain Case (Shenzhen, China).

### CTB Retrograde Tracing

Cholera toxin subunit B (recombinant, CTB) Alexa Fluor‐555 conjugate was obtained from Thermo Fisher (C‐34778). CTB‐555 (200 nl) was delivered stereotactically into the DG (AP, ‐2.0 mm; ML, ‐1.2 mm; DV, ‐2.1 mm). The fusion rate was 50 nL min^−1^; the needle was held for 5 min after the CTB injection. Seven days later, the mice were anaesthetized and perfused.

### Fiber Implantation and Viral Injection Surgery

Similar to our previous studies,^[^ ^43^ ^]^ C57BL/6 mice were mounted in a stereotaxic apparatus under sodium pentobarbital anesthesia (50 mg kg^−1^, i.p.). Virus was injected into the right DG (AP, ‐2.0 mm; ML, ‐1.2 mm; DV, ‐2.1 mm) or the right EC (AP: ‐4.0 mm, ML: ‐4.1 mm, DV: ‐4.8 mm). The above viral suspension was injected with a 1‐µL microliter syringes (Gaoge Industrial and Trading Co. LTD, Shanghai, China) controlled by an injection pump (Micro 2T, World Precision Instruments, USA) at 100 nl min^−1^. After each injection, the needle was left in place for additional 5 min to prevent backflow of the virus and then slowly withdrawn. The virus was allowed to express for a minimum of 4 weeks to achieve sufficient accumulation in the soma and axons. An optical cannula was implanted into the right DG (AP, ‐2.0 mm; ML, ‐1.2 mm; DV, ‐1.9 mm), the right EC (AP, ‐4.0 mm; ML, ‐4.1 mm; DV, ‐4.6 mm) or SuM (AP, −2.4 mm; ML, ‐0.6 mm; DV, −4.5 mm) for optical stimulation and fiber photometry recording. Four screws were placed in the skull over the cortex to secure the dental cement, two of which were used to record EEGs and serve as the reference. All the above coordinates were measured from bregma according to the Paxinos and Franklin's Mouse Brain Atlas^[^ ^44^ ^]^ for mice. The location of optical cannula and viral expression were histologically verified in all mice after the behavioral studies. Only the mice with correct locations of viral expression were taken into analysis.

### Pentylenetetrazol (PTZ) Seizure Model

About 3 weeks after the injection of virus or the termination of EE, a surgery was performed to record EEGs. Two screws were placed separately over the cortex and cerebellum in the skull; the one over the cortex was used to record EEGs, the other over the cerebellum served as the reference electrode. After the surgery, mice were given 7 days of rest and then 20 mg kg^−1^ PTZ (i.p.) initially to induce seizures; an additional dose of 20 mg kg^−1^ PTZ was infused subsequently every 10 min until the mice reached stage 6. EEGs were recorded using a PowerLab 8/35 system (AD Instruments, Australia) for 90 min at a sampling rate of 1 kHz, starting 10 min before PTZ administration. Seizure severity was classified according to a modified Racine scale^[^ ^45^ ^]^ using the following criteria: 1) stage 1 (ear and facial movement), 2) stage 2 (nodding), 3) stage 3 (unilateral forelimb clonus), 4) stage 4 (bilateral forelimb clonus and rearing), 5) stage 5 (falling and jumping with bilateral forelimb clonus), 6) stage 6 (stiffened). Seizure stage 1–3 indicated focal seizures (FSs) and stage 4–6 were general seizures (GSs). The seizure stages were scored by a trained observer who was blinded to the group allocation. For each mouse, seizure stage, latency, number of PTZ doses, percentage of GS and death were recorded and analyzed. Latency to onset was defined as the time required from the injection of the first PTZ to the first appearance of the epileptiform discharge. Epileptiform discharges were defined as a seizure only when they lasted for more than 10 s and had an average spike frequency ≥ 2 Hz.^[^ ^46^ ^]^

To evaluate c‐Fos levels in mGCs, we reduced the number of injections of PTZ: instead of continuously increasing the injections of PTZ until the death of mice, only 3 times of injections were given to each mouse to ensure their survival so that immunostaining of c‐Fos can be performed.

### Optogenetic/Chemogenetic Modulation and Pharmacology

Laser light was delivered through a 200 µm‐diameter optic fiber which was connected to the laser (BL473T3‐050 or YL589T3‐050, Shanghai Laser & Optics Century Co., Ltd., China). Before placing the animal into the chamber, the optic fiber was inserted and secured so as to ensure no movement of the fiber during the whole experiment. In the PTZ model, optical stimulation was delivered during the whole experiment. The blue light (473 nm) stimulation parameter is 473 nm, 20 Hz, 10 ms pulse^−1^ 10s on/ 10s off. For mimicking the effect of EE, optical stimulation was delivered for 7 continuous days. The blue light (473 nm) stimulation parameter is 473 nm, 20 Hz, 10 ms pulse^−1^ 10s on/ 20s off, 15 min. For chemogenetic studies, mice were injected with clozapine‐n‐oxide (CNO) (1.0 mg kg^−1^, i.p., Abcam, ab141704) 30 min before the first injection of PTZ or EE. Control animals were treated identically with CNO; however, they were injected with AAV‐CaMKIIα‐GFP instead of AAV‐CaMKIIα‐hM4Di‐mCherry.

Pharmacological drugs were focally injected into the DG of freely moving mice through the cannula with an inserted injection needle (Catalog No.62004, RWD Life Science, China) connected to a 1‐µL microliter syringes by PE tubing (Catalog No.62320, RWD life science, China). The injection duration for drugs was 2 min and the needle was left in place for another 5 min before withdrawal. For glutamatergic receptor antagonists, we used a blocker cocktail (1:1, 0.5 µL) of 10 µM CNQX (Sigma, C127) and 25 µM D‐AP5 (Abcam, ab120003) dissolved in saline. For the GABA_A_ receptor antagonist, 5 µM bicuculline (0.5 µL) (Abcam, ab120108) dissolved in saline was used. The drugs were injected 5 min before the insertion of optic fiber to deliver blue light. Control animals received saline.

### Fiber Photometry Recording and Analysis

Following GCaMP6m (AAV‐EF1a‐DIO‐GCaMP6m‐WPRE) and pUX‐Cre virus injection, then, a ceramic ferrule (200 µm, 0.37 NA, 6.0 mm, Inper Co. Ltd, Hangzhou, China) was implanted in DG (AP, ‐2.0 mm; ML, ‐1.2 mm; DV, ‐1.8 mm) to record the Ca^2+^ activity. The fiber photometry of calcium signals was carried out in *pUX‐GCaMP6* mice simultaneously with EEG; the photomultiplier tube current output to voltage signals, which were further filtered through a low‐pass filter. Data was further analyzed by MATLAB (version R2020b, MathWorks, USA).

To evaluate the activity of abDGCs in seizures, the signals were recorded for 200 s (100 s baseline and 100 s after a seizure onset). The values of fluorescence change were shown as ΔF/F with the following equation: (ΔF/F) = (F−F_0_)/F_0_, among which F represented the current value of signal, F_0_ represented the average value of baseline signals between 90 and 100 s.

To evaluate the activity of abDGCs in EE, fiber photometry recording was carried out after 4 weeks of injecting AAV2/2Retro‐hSyn‐DIO‐GCaMP6m‐WPRE‐pA into the DG in *CaMKIIα‐Cre* mice. In vivo recordings were carried out in an open‐top home cage or EE containing toys, a running wheel, tunnels, and bridges, for 15 min. The signals were recorded for 20 min (10 min baseline and 10 min during EE). F_0_ represented the average value of baseline signals between 0 and 10 min. The data were presented as time‐related peri‐event plot and heatmap.

### Immunohistochemistry

Mice were deeply anesthetized with pentobarbital sodium and were perfused with cold 0.1 M phosphate‐buffered saline (PBS, pH 7.4 ± 0.1, Cat# 243 176, Biosharp, Anhui, China), followed by cold 4% (w/v) paraformaldehyde (PFA, Cat# P804536, Macklin, Shanghai, China) in PBS. The whole brain was removed and stored in PFA overnight at 4 °C and then dehydrated in 30% sucrose (Cat# S11055, Yuanye Bio‐Technology Co., Ltd, Shanghai, China). Coronal brain sections (25 µm) were frozen in optimum cutting temperature compound (Cat# 6506, Epredia, USA) and cut with a freezing microtome, cryostar NX70 (Thermo Fisher Scientific, CA, USA), then floated in PBS. Sections were blocked with 1% BSA (Cat# A8010, Solarbio, Beijing, China) and 5% donkey serum (Cat# 36116ES10, YEASEN, Shanghai, China) in 0.3% Triton X‐100/PBS for 2 h at room temperature. Free‐floating sections were then incubated overnight at 4 °C with the following primary antibodies: BrdU (1:400, Abcam ab1893), DCX (1:500, Abcam ab18723), c‐Fos (1:500, Abcam ab208942), CaMKIIα (1:500, Abcam ab52476), NeuN (1:500, Proteintech 66836‐1‐Ig), GABA (1:500, Sigma–Aldrich A2052), GFAP (1:500, CST 3670T), Prox1 (1:500, Millipore AB5475) and secondary antibodies Alexafluor 488 (1:500, Abcam ab150073), 594 (1:500, Abcam ab150108) and 647 (1:500, Abcam ab150179). Specially, for immunohistochemical detection of incorporated BrdU, all mice received one injection of BrdU (100 mg kg^−1^, i.p., dissolved in saline, Sigma B5002) during a period of 24 h to label mitotically active cells, and were perfused 72 h after the last injection; tissue was incubated in 2N HCL at 37 °C for 30 min, and then sections were washed in 0.1 M Boric acid pH 8.5 for 5min*3 times to achieve DNA denaturation. Images were captured by confocal microscopes (SP8, Leica and Olympus FV3000). Image analysis and quantification were performed using Image J (version 1.52a) software. For c‐Fos^+^ cell quantification, we counted the number of total cells that exhibited c‐Fos^+^ immunoactivity within the corresponding nucleus of three representative coronal slices (anterior, intermediate, and posterior) of each brain region, and then calculated the mean value for each mouse. The number of mice used in each experiment was indicated in the figure legends. In order to quantify the number of BrdU^+^ or DCX^+^ cells, the labelled cells were counted in a one‐in‐eight series of 30‐µm sections through hippocampus (8 sections per mouse, from anterior to posterior), summed and multiplied by eight to indicate the total number of cells per DG.^[^ ^21^, ^47^ ^]^ To calculate the number of cells showing colocalization of BrdU and DCX, the same method was used as above in constrained sections. The calculation of cells was performed by a trained experimenter who was blind to the group allocation. Figure legends provided information on the number of mice used in each experiment.

### Imaging and the Analysis of Dendrites

Serial coronal sections (25 µm) were prepared using a freezing microtome (Thermo Fisher Scientific, CA, USA), mounted on Superfrost plus slides (Thermo Scientific), stained and coverslipped. For dendrite analysis, abDGCs in the suprapyramidal blade were scanned using a 40× objective with a z‐step size of 1.5 µm. abDGCs were reconstructed by the NeuronStudio software.^[^ ^48^ ^]^ Total dendritic length as well as the number of intersections at concentric circles (20 µm apart) were measured.

### Statistics

Number of experimental replicates (n) was indicated in figure legend. All data were presented as the mean ± S.E.M. Student's t‐test or Mann Whitney test was used to compare the differences between two groups; one‐way analysis of variance (ANOVA) followed by Tukey's post hoc test or Kruskal‐Wallis test was used for multiple group comparisons. Two‐way ANOVA followed by the Bonferroni's post hoc test or Sidak's test was used was used for two group comparisons. Log‐rank (Mantel‐Cox) tests were used to compare whole curves. Statistical comparisons were performed using Prism (version 7.0) with appropriate inferential methods as indicated in the figure legends. A two‐tailed P value < 0.05 was considered statistically significant (*p<0.05, **p<0.01, ***p<0.001 and ****p<0.0001).

## Conflict of Interest

The authors declare no conflict of interest.

## Author Contributions

Z.L., L.C. and F.F. contributed equally to this work. Z.C., Y.W., Z.X.L. and L.Y.C. designed the study and wrote the manuscript. Y.W., Z.X.L., L.Y.C., L.Y. and F.F. analyzed the data. Z.X.L., Y.W. and Y.W.X performed the behavioral tests. Z.X.L., L.Y.C. and W.Q.W. contributed to the molecular biological experiments. Z.X.L., W.Q.W., H.M.C and F.F. contributed to mouse breeding. C.L.X, S.W., Y.G. F.H. and all other authors contributed to discussion.

## Supporting information



Supporting Information

## Data Availability

The data that support the findings of this study are available from the corresponding author upon reasonable request.
