# Supplementary material for: Enriched Environment Reduces Seizure Susceptibility via Entorhinal Cortex Circuit Augmented Adult Neurogenesis
Source: Adv Sci (Weinh). 2024 Oct 22;11(46):2410927. doi: 10.1002/advs.202410927 (PMC11633471; doi:10.1002/advs.202410927)
Supplement: Supplementary file 1 — Supporting Information [file ADVS-11-2410927-s001.pdf]

ADVANCED SCIENCE
Open Access

Supporting Information

for *Adv. Sci.*, DOI 10.1002/advs.202410927

Enriched Environment Reduces Seizure Susceptibility via Entorhinal Cortex Circuit Augmented Adult Neurogenesis

Zhongxia Li, Liying Chen, Fan Fei, Wenqi Wang, Lin Yang, Yu Wang, Heming Cheng, Yingwei Xu, Cenglin Xu, Shuang Wang, Yan Gu, Feng Han, Zhong Chen and Yi Wang**

Supplementary Information

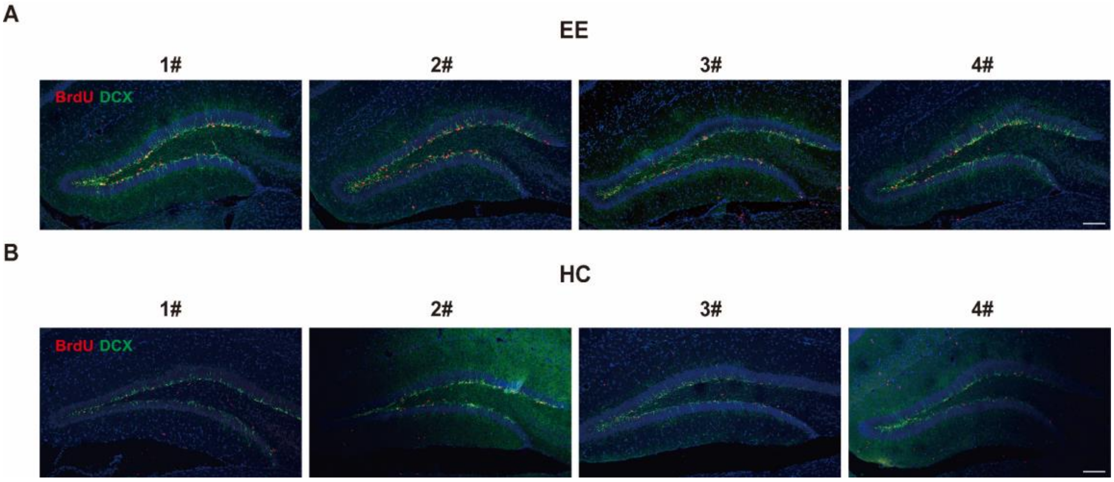

Figure S1 Immunohistochemistry of BrdU and DCX expression for each individual HC and EE mice.

(**A**) EE group (n=4). (**B**) HC group (n=4).

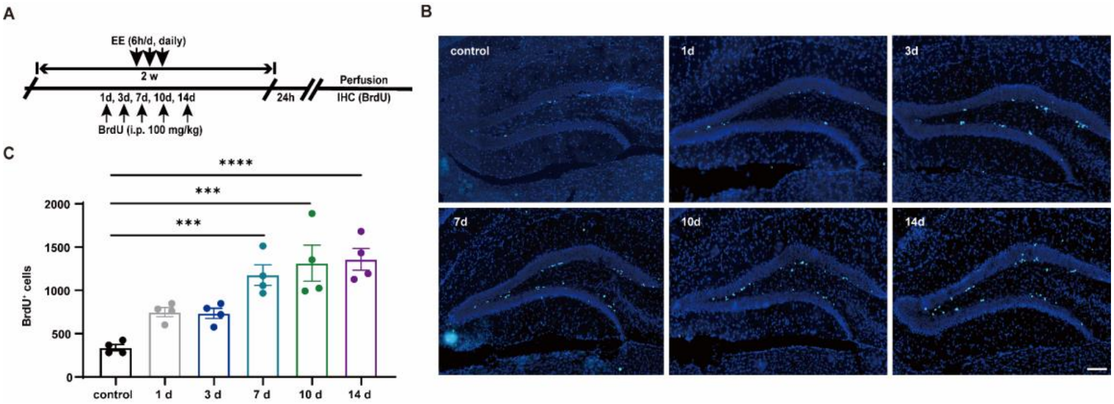

Figure S2 The number of BrdU-positive cells is significantly increased with longer EE treatment time.

(**A**) Experiment scheme of using BrdU to label cell proliferation in adult SGZ at different timepoints during EE treatment. BrdU was administered once daily (100mg/kg) at 1d, 3d, 7d, 10d, 14d separately and mice were sacrificed 24h after the injection of BrdU. (**B**) Representative immunohistochemistry images of BrdU labeling at different timepoints during EE treatment (bar = 100 μm). (**C**) Proliferative activity in the SGZ was significantly increased with longer EE treatment time (n = 4 for each group, ***$p < 0.001$, ****$p < 0.0001$, compared with control; One-way ANOVA with post hoc Tukey's test).

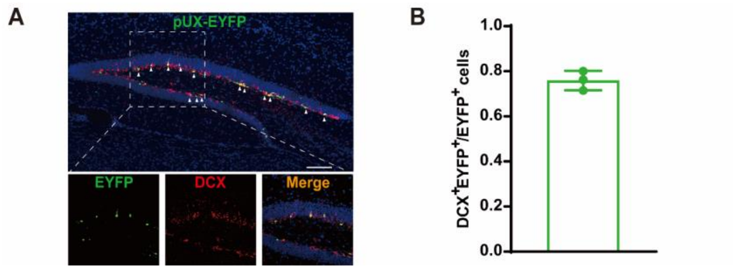

Figure S3 Immunohistochemistry of DCX in EYFP-expressing abDGCs labelled with our dual virus strategies.

(**A**) Representative images of double immunostaining of EYFP (EYFP) with DCX (red) (bar = 100 μm). Mice were perfused 3 days after the injection of dual virus (pUX-Cre plus DIO-EYFP). (**B**) The percentage of DCX^+ in $EYFP^+$ cells (n=3, 75.87%).

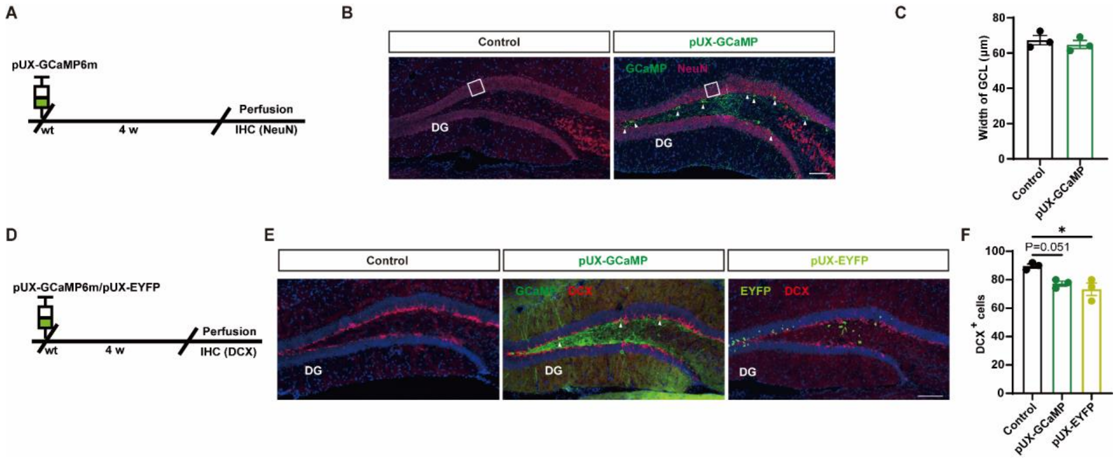

Figure S4 A dual virus strategy with cocktail of AAV and pUX-Cre (retrovirus) results in a slight reduction in the number of newborn abDGCs.

(**A**) Experimental scheme of immunostaining with NeuN. 4 weeks after dual virus injection into DG, mice were perfused and immunohistochemistry was conducted. (**B**) Representative images of expression of GCaMP (EYFP) and NeuN (Red) (bar = 100 µm). (**C**) The width of granule cell layer (GCL) was calculated and compared (n=3 for each group; Student's t-test). (**D**) Experimental scheme of immunostaining with DCX. 4 weeks after dual virus injection into DG, separately pUX-Cre plus DIO-GCaMP6m and pUX-Cre plus DIO-EYFP, mice were perfused and immunohistochemistry was conducted. (**E**) Representative images of GCaMP (EYFP) and DCX (Red) (bar = 100 µm). (**F**) DCX expression was slightly reduced after the injection of a viral cocktail of AAV and pUX-Cre (retrovirus) (n=3 for each group, *$p<0.05$; One-way ANOVA with post hoc Tukey's test).

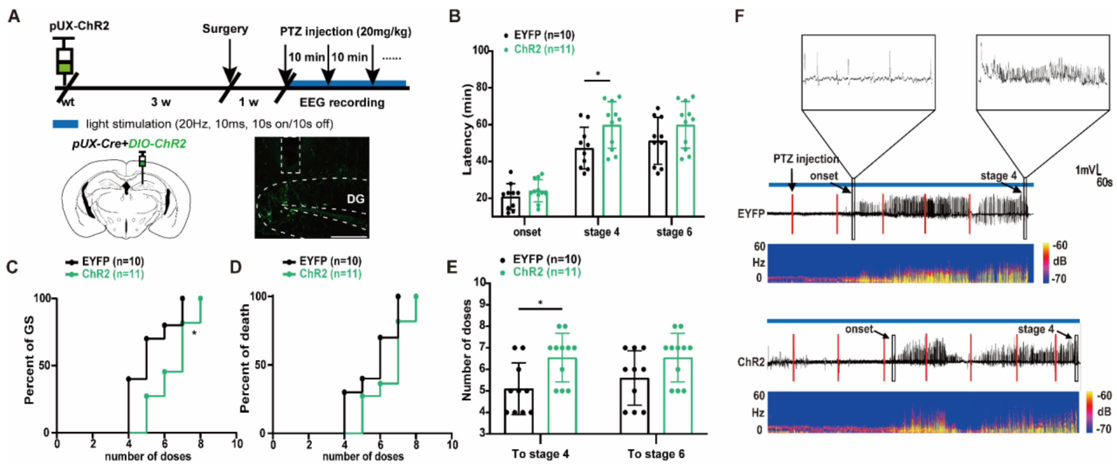

Figure S5 Optogenetic activation of abDGCs reduces seizure susceptibility.

(**A**) Experiment scheme for optogenetic activation of the abDGCs in a PTZ-induced seizure model. Blue light stimulation was given throughout the behavioral tests. (**B-E**) Effects of abDGCs on seizure susceptibility in a PTZ-induced seizure model; (**B**) latency to onset, stage 4 and stage 6; (**C**) percent of mice reaching GS with increasing number of doses; (**D**) percent of death with increasing number of doses; (**E**) number of doses to stage 4 and stage 6. n=10 for EYFP, n=11 for ChR2, *$p<0.05$; for **B, E** Two-way ANOVA followed by Sidak's test; for **C, D** Log-rank (Mantel-Cox) tests were used to compare whole curves. (**F**) Typical EEGs and power spectrograms recorded from the cortex during seizures in a PTZ induced seizure model; the horizontal blue bar indicates the time for delivery of blue light.

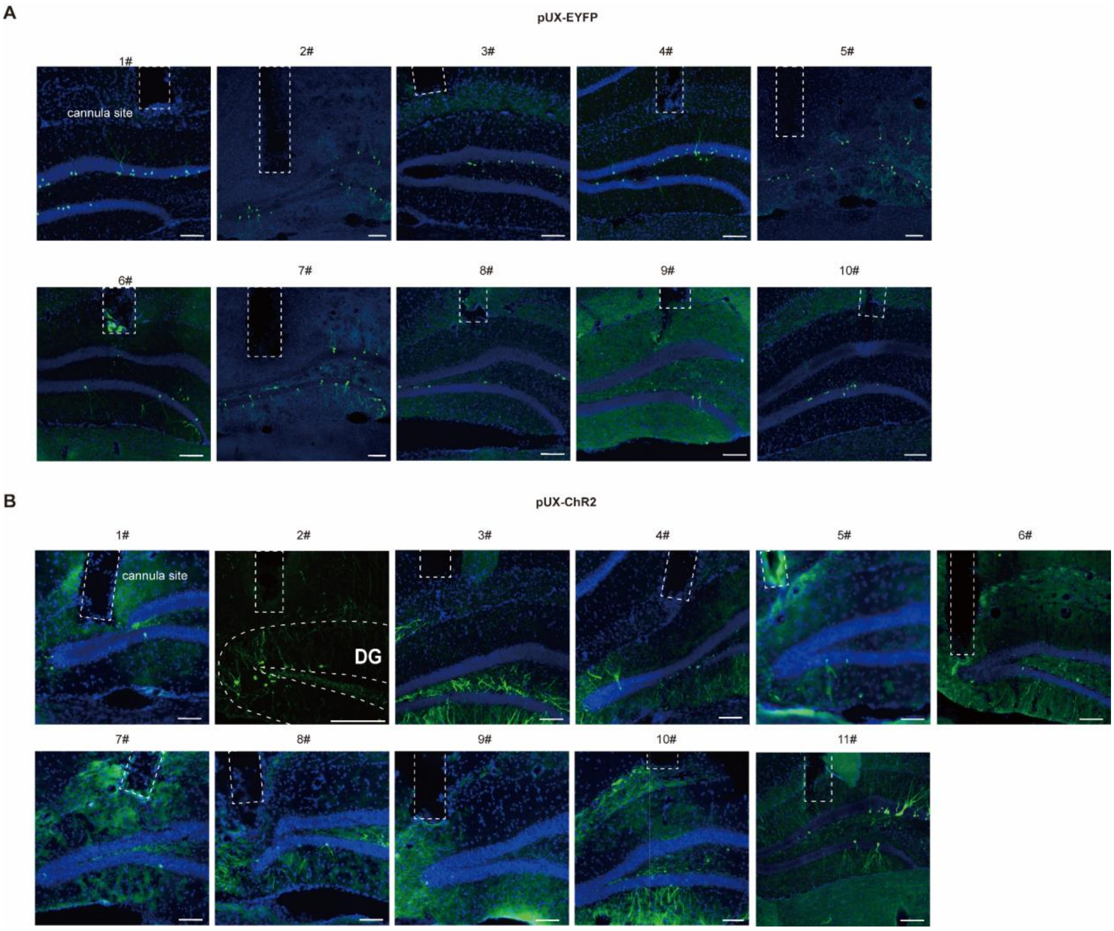

Figure S6 Representative DIO-EYFP and DIO-ChR2 expression for individual mouse in Fig S5.

(**A**) Mice were injected with pUX-Cre and DIO-EYFP to serve as control group**.** (**B**) Mice were injected with pUX-Cre and DIO-ChR2. The image of mouse (2#) was the one we selected to represent the virus expression and optic location of the "optogenetic activation (ChR2) group" in Figure S5.

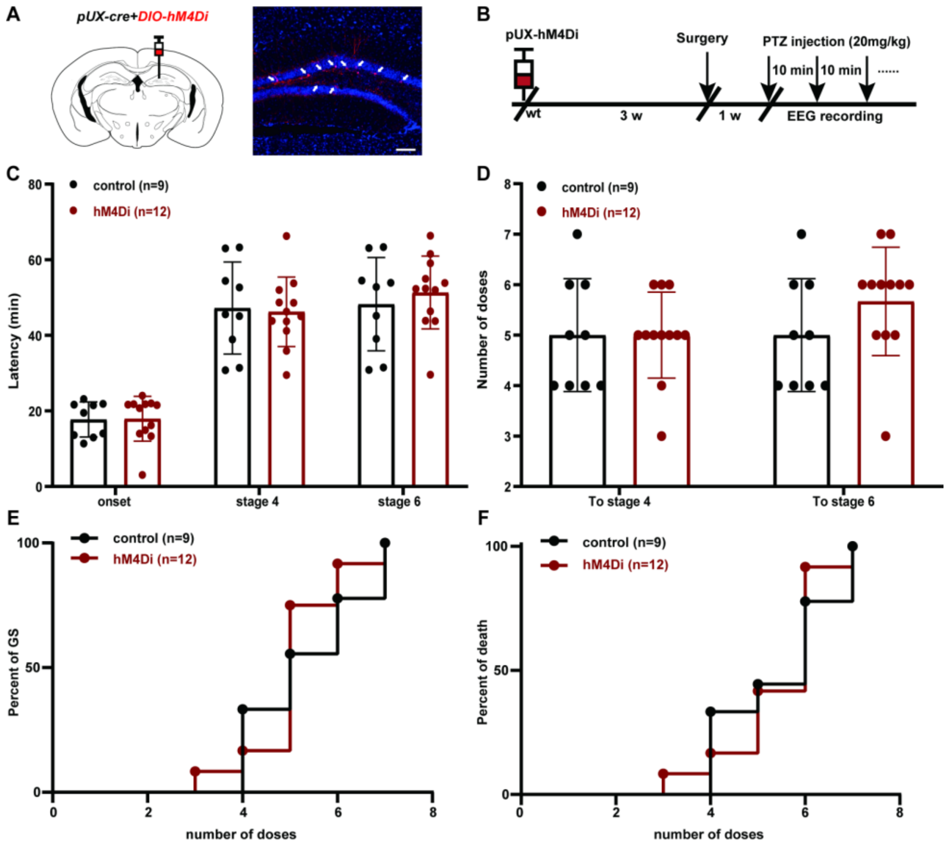

Figure S7 Chemogenetic inhibition of abDGCs born at basal state has no effect on seizure susceptibility.

(**A**) Immunostaining of hM4Di (red) expression in the DG. White arrows point to the labelled abDGCs (bar = 100 μm). (**B**) Experiment scheme for chemogenetic inhibition of the abDGCs in a PTZ-induced seizure model. CNO was injected (1.0 mg/kg, i.p.) 30 min before the first injection of PTZ. (**C-F**) Effects of abDGCs on seizure susceptibility in a PTZ-induced seizure model; (**C**) latency to onset, stage 4 and stage 6; (**D**) number of doses to stage 4 and stage 6; (**E**) percent of mice reaching GS with increasing number of doses; (**F**) percent of death with increasing number of doses. n=9 for mCherry, n=12 for hM4Di. For **C**, **D** Two-way ANOVA followed by Sidak's test; for **E**, **F** Log-rank (Mantel-Cox) tests were used to compare whole curves.

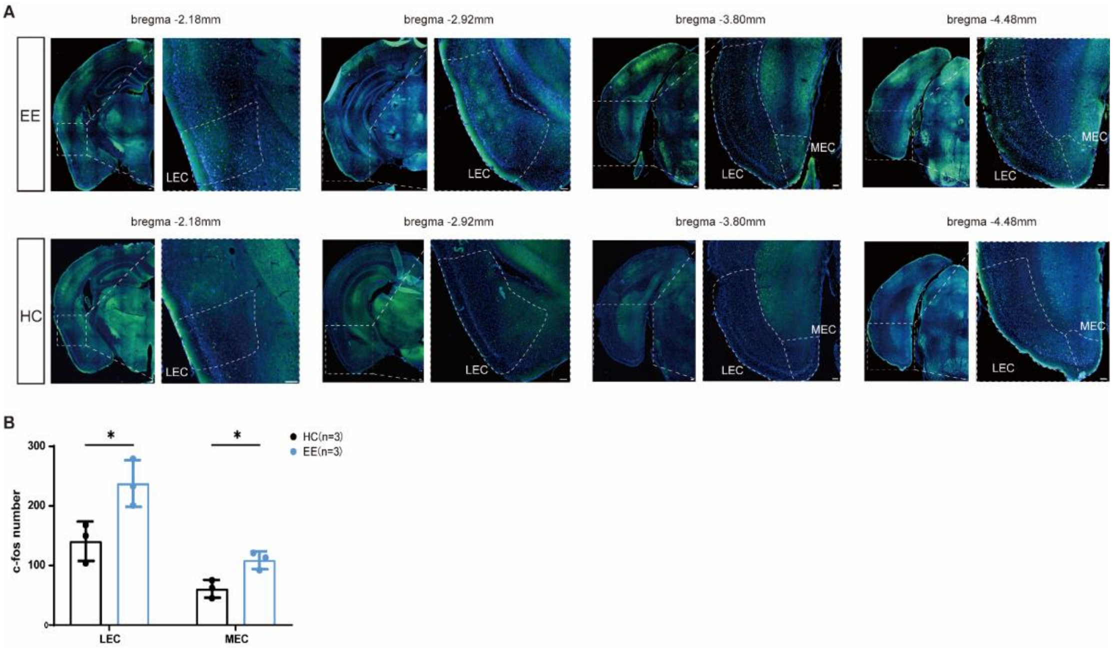

Figure S8 EE significantly increases the number of c-Fos-positive cells in both MEC and LEC.

(**A**) Representative images indicating the distribution of c-Fos$^+$ neurons in LEC and MEC (bar = 100 μm). (**B**) The number of c-Fos$^+$ neurons in LEC and MEC of mice housed in HC (control) and after EE treatment. (n=3 for each group *p<0.05, compared with control; Two-way ANOVA followed by Sidak's test).

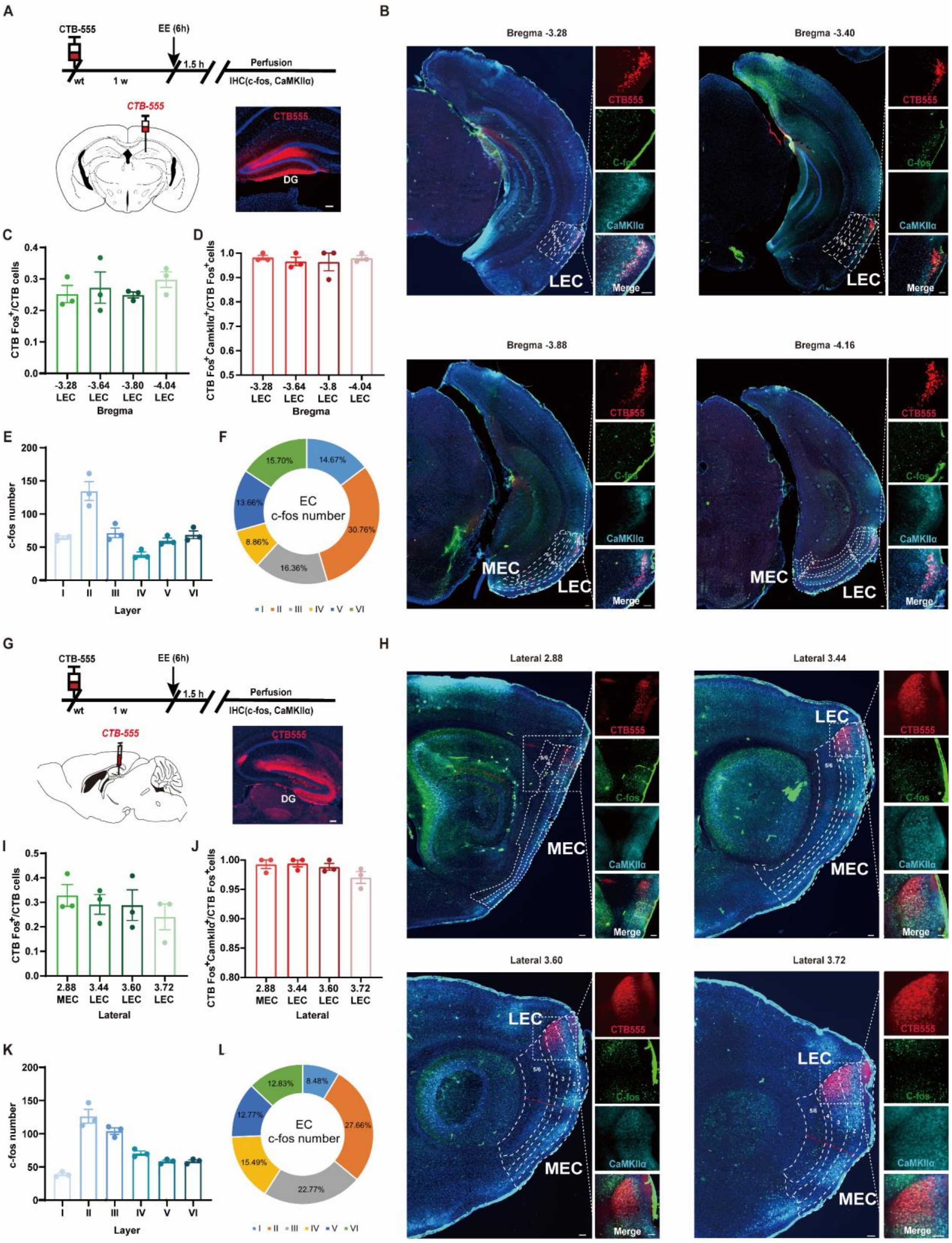

Figure S9 DG-projecting EC neurons show layer-dependent activation after EE treatment.

(**A**) Experiment scheme of c-Fos labeling in response to EE in DG-projecting EC neurons (coronal slices). CTB-555 was injected into DG (bar = 100 μm) to visualize DG-projecting neurons. (**B**) Representative photomicrographs from coronal slices indicating the distribution of c-Fos^{+} DG-projecting CaMKIIα^{+} neurons in EC (bar = 100 μm). (**C**) The percentage of c-Fos^{+} in DG-projecting LEC neurons (n=3 for each group; One-way ANOVA with post hoc Tukey's test). (**D**) The percentage of CaMKIIα^{+} in c-Fos^{+} DG-projecting LEC neurons (n=3 for each group; One-way

ANOVA with post hoc Tukey's test). (**E**) The number of c-Fos^+ neurons in layer-I to layer VI of EC, respectively, after EE treatment. (n=3 for each group). (**F**) Layer-II has the most percentage (30.76%) of c-Fos^+ neurons. (**G**) Experiment scheme of c-Fos labeling in response to EE in DG-projecting EC neurons (sagittal slices). CTB-555 was injected into DG (bar = 100 μm) to visualize DG-projecting neurons. (**H**) Representative photomicrographs from sagittal slices indicating the distribution of c-Fos^+ DG-projecting CaMKIIα^+ neurons in EC (bar = 100 μm). (**I**) The percentage of c-Fos^+ in DG-projecting LEC/MEC neurons (n=3 for each group; One-way ANOVA with post hoc Tukey's test). (**J**) The percentage of CaMKIIα^+ in c-Fos^+ DG-projecting LEC/MEC neurons (n=3 for each group; One-way ANOVA with post hoc Tukey's test). (**K**) The number of c-Fos^+ neurons in layer-I to layer VI of EC, respectively, after EE treatment (n=3 for each group). (**L**) Layer-II has the most percentage (27.66%) of c-Fos^+ neurons.

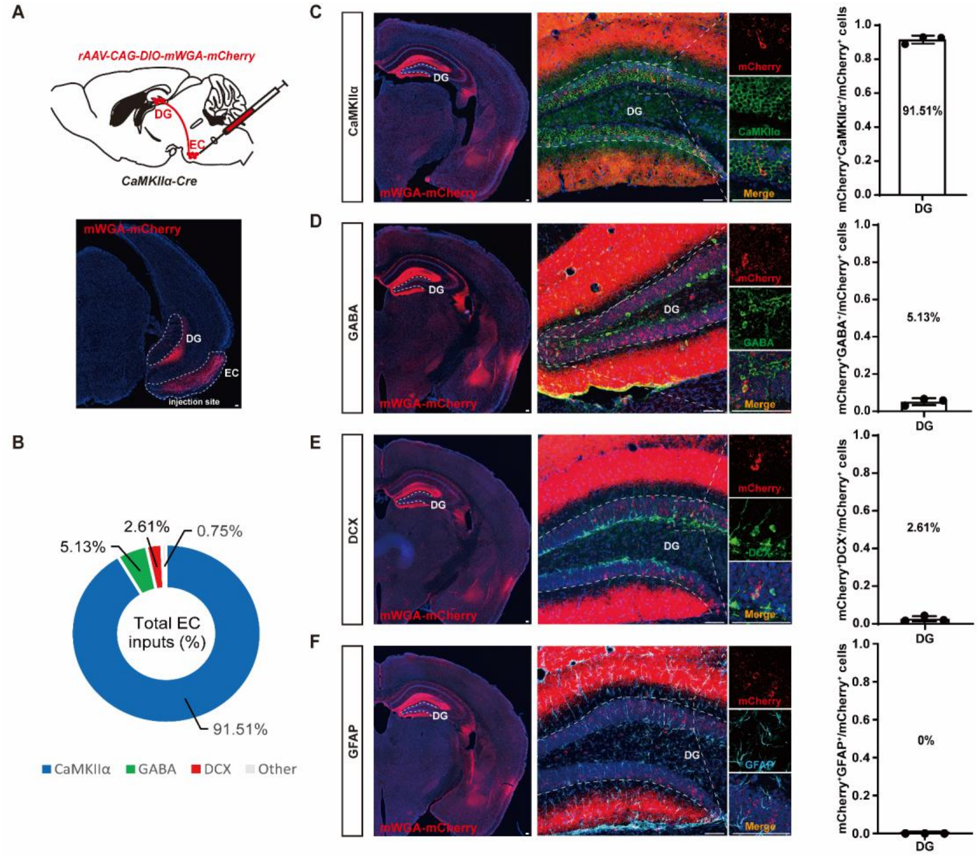

Figure S10 The cellular target of the projections from EC CaMKIIα$^+$ neurons to DG.

(**A**) Experimental scheme for injection of antegrade transsynaptic virus to map monosynaptic outputs from the EC CaMKIIα$^+$ neurons (upper panel). rAAV-CAG-DIO-mWGA-mCherry was injected into the EC of *CaMKIIα-Cre* transgenic mice. Representative images of the injection site in EC (bar=100 μm) (lower panel). (**B**) Pie chart indicates the percentage of DG cells receiving direct outputs from EC CaMKIIα$^+$ neurons while colocalizing with separately CaMKIIα (91.51%), GABA (5.13%), DCX (2.61%). (**C-F**) Representative images of DG cells receiving output from EC CaMKIIα$^+$ neurons while colocalizing with CaMKIIα (**C**), GABA (**D**), DCX (**E**) and GFAP (**F**), respectively (n=3, bar=100 μm).

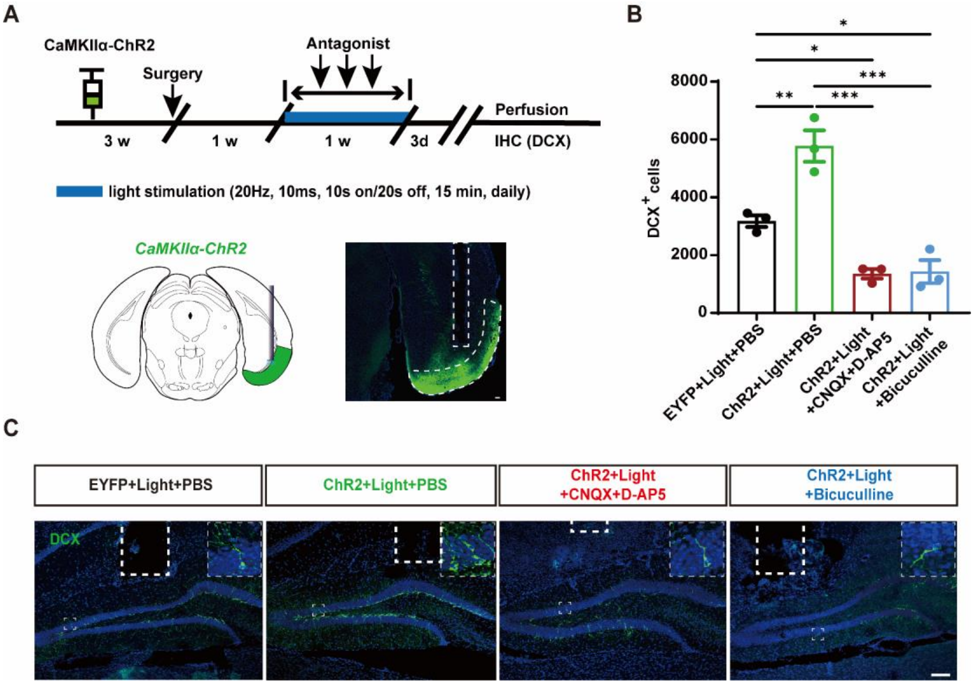

Figure S11 The application of glutamate antagonists or GABA antagonists in DG abolishes the effect of EC-activation induced AHN-upregulating.

(**A**) Upper: Experiment scheme for administration of pharmacological blockades while optogenetic stimulating EC CaMKIIα^{+} neurons. Glutamate antagonists (intra-DG injection, CNQX10 μM plus D-AP5 25 μM, 0.5μL) and the $GABA_A$ receptor antagonist bicuculline (intra-DG injection, 5μM, 0.5μL) were administered 10 min before optogenetic stimulation. Lower: Representative immunostaining of ChR2 (green) expression in the EC (bar = 100 μm). (**B**) Proliferative activity in the SGZ was significantly reduced after the application of glutamate antagonists or GABA antagonists (n=3 for each group *p<0.05, **p<0.01, ***p<0.001; One-way ANOVA with post hoc Tukey's test). (**C**) Representative images of DCX^{+} cells in the DG (bar = 100 μm).

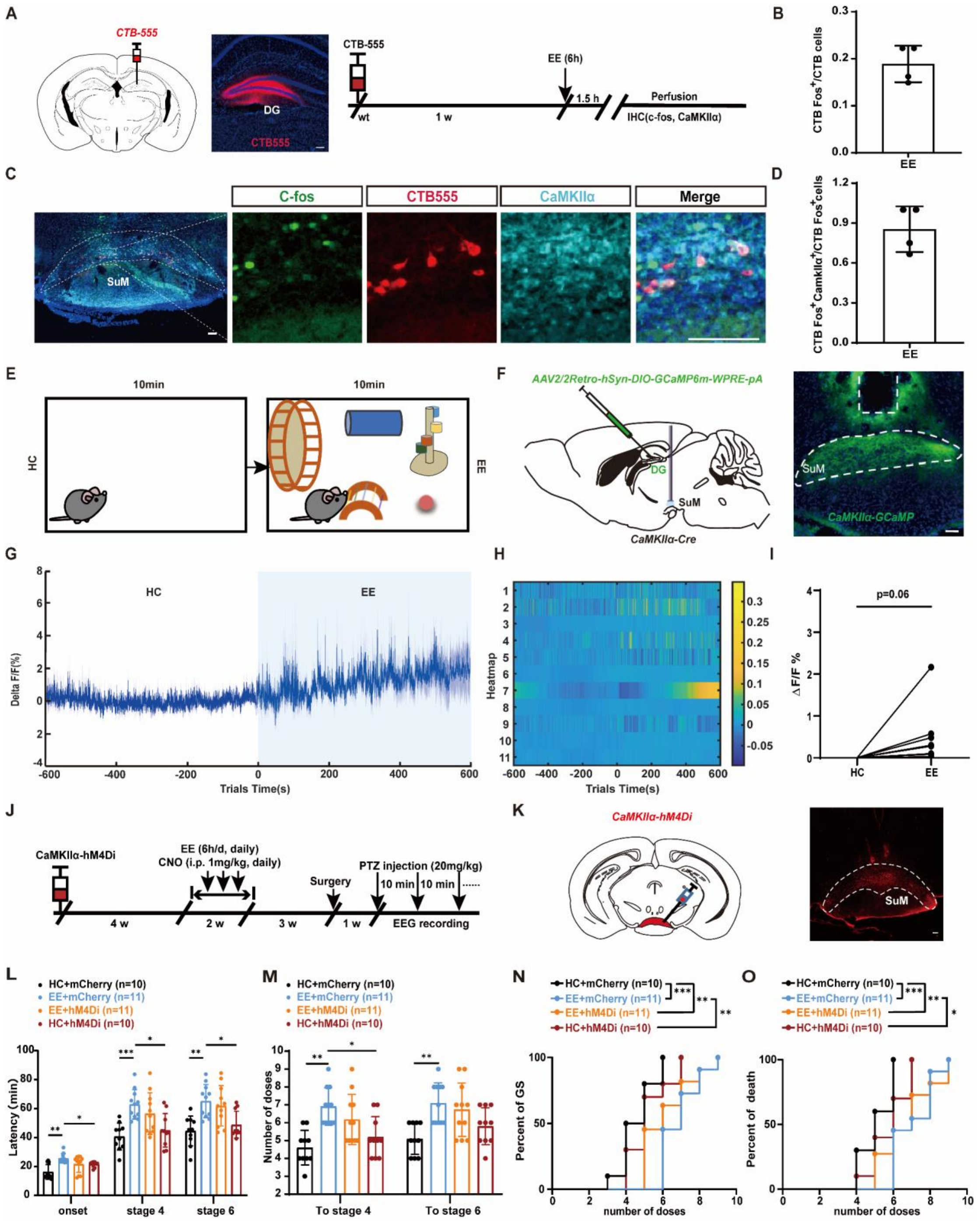

Figure S12 DG-projecting SuM CaMKIIα⁺ neurons are not required for anti-seizure effect of EE treatment.

(**A**) Experiment scheme of c-Fos expression in response to EE in DG-projecting SuM neurons. CTB-555 was injected into DG (bar = 100 μm) to visualize DG-projecting neurons. Mice were perfused 1.5 h after the completion of EE treatment and immunohistochemistry was then performed. (**B**) The percentage of c-Fos$^+$ in DG-projecting SuM neurons (n=4). (**C**) Representative photomicrographs indicating the distribution of c-Fos$^+$ DG-projecting CaMKIIα$^+$ neurons in SuM (bar = 100 μm). (**D**) The percentage of CaMKIIα$^+$ in DG-projecting SuM c-Fos$^+$ neurons (n=4). (**E**) Diagram of in vivo fiber photometry recording of free-moving mice in home cage

(HC) and EE for 10 min. (**F**) Left: Diagram of calcium recording of DG-projecting SuM neurons. AAV2/2Retro-DIO-GCaMP6m-WPRE-pA was injected into the DG of *CaMKIIα-Cre* mice to express GCaMP6 onto DG-projecting CaMKIIα$^+$ neurons and the optical fiber was inserted right above SuM to collect calcium signals. Right: Representative images of GCaMP6m expression in SuM neurons and the location of optic fiber (bar = 100 μm). (**G**) Mean fluorescence values of population activity of DG-projecting SuM neurons in the HC and EE. (**H**) Heatmaps showing change of calcium signals. (**I**) The statistical value of $\triangle F/F_0$ was shown for each mouse (n=11, Paired t-tests). (**J**) Experiment scheme for chemogenetic inhibition of SuM CaMKIIα$^+$ neurons during EE treatment in a PTZ-induced seizure model. CNO was administrated for 14 days during EE treatment (30 min before EE, i.p. 1.0 mg/kg, daily). (**K**) Immunostaining of hM4Di (red) expression in the SuM (bar = 100 μm). (**L-O**) Effects of chemogenetic inhibition of EC CaMKIIα$^+$ neurons in EE on seizure susceptibility in a PTZ-induced seizure model; (**L**) latency to onset, stage 4 and stage 6; (**M**) number of doses to stage 4 and stage 6; (**N**) percent of death with increasing number of doses; (**O**) percent of mice reaching GS with increasing number of doses. n=10 for HC+mCherry and HC+hM4Di group, n=11 for EE+mCherry and EE+hM4Di group. For **L**, **M** Two-way ANOVA followed by Tukey's test; for **N**, **O** Log-rank (Mantel-Cox) tests were used to compare whole curves.

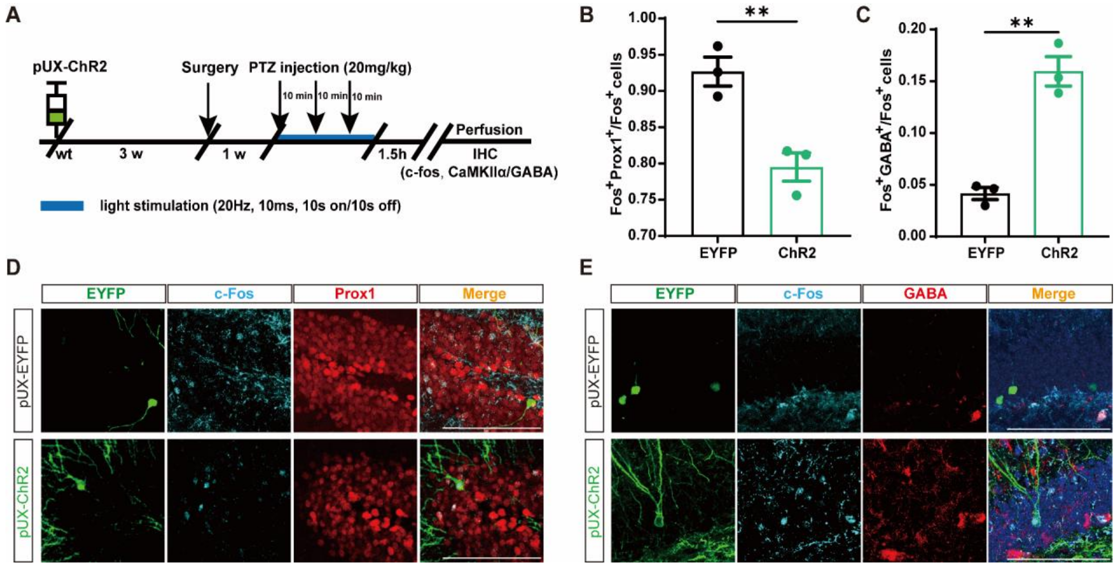

Figure S13 Optogenetic activation of abDGCs reduces c-Fos expression in DG Prox1$^+$ neurons and increases c-Fos expression of DG GABA$^+$ neurons in a PTZ-induced seizure model.

(**A**) Experimental scheme for optogenetic activation of abDGCs in a PTZ-induced seizure model. Mice were perfused 1.5 h after the end of the light stimulation. (**B**) The percentage of Prox1$^+$ in c-Fos$^+$ neurons (n=3 for each group **p<0.01; Student's t-tests)**.** **(C)** The percentage of GABA$^+$ in c-Fos$^+$ neurons (n=3 for each group **p<0.01; Student's t-tests). (**D-E**) Representative images indicating the distribution of Prox1$^+$ c-Fos$^+$ neurons **(D)** and GABA$^+$ c-Fos$^+$ neurons **(E)** in DG (bar = 100 μm).
